# Quantitative Analysis of Fragrance and Odorants Released from Fresh and Decaying Strawberries

**DOI:** 10.3390/s130607939

**Published:** 2013-06-20

**Authors:** Yong-Hyun Kim, Ki-Hyun Kim, Jan E. Szulejko, David Parker

**Affiliations:** 1 Atmospheric Environment Laboratory, Department of Environment & Energy, Sejong University, Seoul 143-747, Korea; E-Mails: inocent01@nate.com (Y.-H.K.); jan.szulejko@btinternet.com (J.E.S.); 2 Palo Duro Research Center, West Texas A&M University, Canyon, TX 79016, USA; E-Mail: dparker@mail.wtamu.edu

**Keywords:** fresh and decaying strawberry, strawberry fragrances, mass concentration, threshold, odor activity value (OAV)

## Abstract

The classes and concentrations of volatile organic compounds (VOC) released from fresh and decaying strawberries were investigated and compared. In this study, a total of 147 strawberry volatiles were quantified before and after nine days of storage to explore differences in the aroma profile between fresh strawberries (storage days (SRD) of 0, 1, and 3) and those that had started to decay (SRD = 6 and 9). In terms of concentration, seven compounds dominated the aroma profile of fresh strawberries (relative composition (RC) up to 97.4% by mass, sum concentration): (1) ethyl acetate = 518 mg·m^−3^, (2) methyl acetate = 239 mg·m^−3^, (3) ethyl butyrate = 13.5 mg·m^−3^, (4) methyl butyrate = 11.1 mg·m^−3^, (5) acetaldehyde = 24.9 mg·m^−3^, (6) acetic acid = 15.2 mg·m^−3^, and (7) acetone = 13.9 mg·m^−3^. In contrast, two alcohols dominated the aroma profile of decayed samples (RC up to 98.6%): (1) ethyl alcohol = 94.2 mg·m^−3^ and (2) isobutyl alcohol = 289 mg·m^−3^. Alternatively; if the aroma profiles are re-evaluated by summing odor activity values (ΣOAV); four ester compounds ((1) ethyl butyrate (6,160); (2) ethyl hexanoate (3,608); (3) ethyl isovalerate (1,592); and (4) ethyl 2-methylbutyrate (942)) were identified as the key constituents of fresh strawberry aroma (SRD-0). As the strawberries began to decay; isobutyl alcohol recorded the maximum OAV of 114 (relative proportion (RP) (SRD = 6) = 58.3%). However, as the decay process continued, the total OAV dropped further by 3 to 4 orders of magnitude—decreasing to 196 on SRD = 6 to 7.37 on SRD = 9. The overall results of this study confirm dramatic changes in the aroma profile of strawberries over time, especially with the onset of decay.

## 1. Introduction

Strawberries are one of the most widely consumed fruits with a good flavor and high nutritional value [1,2]. The aroma and odor-quality of strawberries depend on the type and concentration of volatile hydrocarbons (HC) in the aroma profile [3–5]. In fact, it is estimated that more than 360 volatile organic compounds (VOC) are emitted from strawberries [6,7]. However, only a small number of these contribute significantly to the strawberry fragrance and impact its perceived quality [8]. The intensity of strawberry fragrance has also been found to vary with the degree of freshness of the fruit. If the characteristics of VOC emitted from strawberries are evaluated thoroughly over time, this information can be used to improve our understanding of the natural strawberry fragrance and allow growers and retailers to optimize their harvesting, packing, storage and retail display procedures.

In order to assess the VOCs released from strawberry, researchers have used several different analytical approaches. Gas chromatographs (GCs) equipped with either flame ionization (FID) or mass spectrometer (MS) detectors have been the most common choices [9–11]. Recently, strawberry fragrances were also evaluated by combining olfactometry and GC techniques—*i.e.*, harnessing state-of-the-art analytical technology alongside the particular selectivity of the human nose [1,2,12].

In this study, the concentrations and chemical types of strawberry volatiles were analyzed to characterize the fragrance (aroma profile: freshness staging) and offensive odorants (due to decay). All volatile compounds released from strawberry samples were collected at five different intervals (up to 9 days of storage period) at 25 °C. For the quantification of volatile components, liquid-phase standard was prepared containing a total of 19 odorous compounds for external calibration (Table 1S) The numbering of all supplementary (S) Tables and Figures are made with an S symbol following the number and placed in the Appendix section at the end. These calibration results were then used to develop predictive equations based on effective carbon number (ECN) [13]. These equations were then used for an extensive list of ‘compounds lacking authentic standards/surrogates (CLASS)’ due to the absence of standard material (*i.e.*, authentic compounds) or to the synthesis complexities or costs involved in standard preparation [14]. The use of the predictive equations based on response factor *vs.* effective carbon number (ECN) linear correlation allowed robust, statistical estimation of all CLASS. The results of this approximation method allowed us to characterize the emission pattern of most fragrance and odorous components released from strawberry samples in a quantitative manner. In this research, we undertook measurements of strawberry aromas and odorants to provide detailed descriptions on their emission patterns in relation to storage duration. The results of this study will thus help us understand the characteristics of the flavor changes in strawberries that occur during storage.

## 2. Materials and Methods

In this research, a total of 19 VOCs that had relatively strong odor intensities with a wide range of volatility and polarity were selected for external calibration (Table 1S). The calibration results obtained using this standard mixture was used to derive predictive equations based on ‘effective carbon number (ECN)’ theory [13]. These ECN-based predictive equations were then used to calculate the concentrations of ‘CLASS’ due to the absence of standard material (*i.e.*, authentic compounds) or to the complexity involved in standard preparation [14].

Liquid-phase working standards (L-WS) of 19 VOCs in methanol were prepared to include: (1) five aldehydes: acetaldehyde (AA), propionaldehyde (PA), butyraldehyde (BA), isovaleraldehyde (IA), and *n*-valeraldehyde (VA), (2) six aromatics hydrocarbons: benzene (B), toluene (T), styrene (S), *p*-xylene (p-X), *m*-xylene (m-X), and *o*-xylene (o-X), (3) two ketones: methyl ethyl ketone (MEK) and methyl isobutyl ketone (MIBK), (4) one alcohol: isobutyl alcohol (i-BuAl), (5) one ester: *n*-butyl acetate (BuAc), and (6) four volatile fatty acids: propionic acid (PPA), butyric acid (BTA), isovaleric acid (IVA), and n-valeric acid (VLA) (Table 1S). The detailed procedures to make the L-WS are described in Table 2S.

The concentrations of CLASS were derived from the predictive equations based on linear regression equations between RF values of target standard compounds (Table 3S) and their effective carbon numbers (ECNs). The ECN was determined by counting the number of the atoms (C, H, and O) and moieties in functional groups (e.g., ether, carbonyl, and methyl groups) in terms of ‘carbon number equivalent (CNE)’ in light of their approximate relative contribution to the sensitivity (RF) in the MS system: ECN = I × (CNE of C) + J × (CNE of H) + K × (CNE of O) + (CNE of > C = O) + M × (CNE of -O-) + N × (CNE of -CH_3_) (Figure S1): (1) C = 1, (2) H = -0.035, (3) O = 0, (4) >C = O = -0.95, (5) -O- = 0.55, and (6) -CH_3_ = 0.15). As 10 out of 147 volatiles detected from the strawberry samples matched with 19 VOCs contained in the L-WS, they were quantified directly using the calibration data of the L-WS. However, we did not prepare standards for the remaining 137 volatiles for many different reasons. For simple quantitation of those strawberry aroma components, we treated them as CLASS to quantify their concentrations based on the ECN approach (Table 4S).

### 2.1. Approaches for the Collection of Volatile Components and Instrumental Setup

#### 2.1.1. The Collection of Strawberry Volatiles

The sorbent tube sampling method was used for the collection of the VOCs released from the cut strawberry sample. In the case of ammonia and RSC, the bag sampling method (polyester aluminum-PEA bag) was used as discussed in the next subsection. The sorbent tube was prepared as a three-bed sorbent by packing with 100 mg of Tenax TA, Carbopack B, and Carboxen 1000 (Supelco, Bellefonte, PA, USA) in a SS tube holder (tube size: length: 9 cm, OD: 6 mm, and ID: 5 mm; Camsco, Houston, TX, USA). The strawberries for this study were grown in Jin Ju city, Gyeong Sang Nam Do Province, Korea. The strawberries (1 kg in a Styrofoam tray) were purchased from a local market within one day after harvesting. An approximately 50 gram of strawberry sample was sliced and placed inside a 750 mL capacity impinger with the gas inlet and outlet positioned as the side arm and nozzle cap, respectively (ID: 3 mm and length: 30 mm). The strawberry samples were cut into four pieces (3 cm ×3 cm × 5.5 cm) (The weight of sample was adjusted to ∼50 g) (Figure S2). The inlet and outlet of the impinger were connected to a 10 L PEA bag filled with back-up gas (ultra-pure nitrogen > 99.999%) and the inlet of the sorbent tube (ST), respectively. A Teflon tube was used to connect the impinger and the PEA bag at one end and the ST at the other. The outlet of the ST was connected to the mini vacuum pump interfaced with mass flow controller (MFC) (Shibata ΣMP-30, Saitama, Japan) using silicon tubing. The VOCs emitted from the strawberry were flushed onto the ST by pumping the nitrogen gas through the impinger containing the strawberry samples at a flow rate of 50 mL·min ^−1^ for 1 min. The impinger was maintained at 25 °C using a temperature-controlling water bath (Figure 1). VOCs emitted from the strawberry sample were collected five times during the entire storage period of 9 days set for this study at 0, 1, 3, 6 and 9 days (Table 5S). For each selected day, the collection of samples was made as replicate for each target group (VOC, RSC, ammonia, and olfactory analysis). To initiate each sampling at a given day, a pre-purge was conducted by supplying ultra-pure nitrogen into the impinger at a flow rate of 50 mL·min ^−1^ for 20 min. Throughout the storage period, the strawberry sample in the impinger was maintained in an aerobic state as the inlet and outlet of the impinger were left open to the air in the laboratory.

#### 2.1.2. Instrumental Setup for VOC Analysis

All the analyses in this study were carried out using a GC-2100 (Shimadzu, Kyoto, Japan) equipped with a QP2010 MS (Shimadzu) and a UNITY thermal desorber (TD: Markes International, Ltd, Llantrisant, UK). The TD focusing cold trap was packed with Tenax TA and Carbopack B in a 1:1 volume ratio (inner diameter = 2 mm and total sorbent bed length: 50 mm). The VOCs were separated on a CP Wax column (diameter = 0.25 mm, length = 60 m, and film thickness = 0.25 μm) using a 50 min GC analytical cycle. The separated VOCs were detected by MS system and identified through library searching (NIST mass spectral library, NIST, Gaithersburg, MD, USA). The detailed conditions are also presented in Table 5S.

### 2.2. Calibration of the Liquid Working Standards of VOCs

Five-point calibration curves were prepared by analyzing sorbent tubes loaded with 1 μL of L-WS at these different concentration levels: (1) 1.30, (2) 6.52, (3) 13.0, (4) 26.1, and (5) 65.2 ng·μL^−1^) (Table 2S). Details of the approach used to introduce the liquid standards to the sorbent tubes in the vapor phase have been described in detail elsewhere [15]. In short, a micro-syringe was used to introduce the liquid standard into the sampling end of the ST in a 50 mL·min^−1^ flow of nitrogen for 10 minutes. Each loaded sorbent tube was then analyzed by the TD-GC-MS system described below.

The sensitivity of the instrumental system remained fairly constant leading to stable response factors throughout the 9-day study period (Table 3S). All coefficients of variation (CV (%): SD/mean × 100) for the RF values were fairly stable (CV = 1.46 ± 1.29% (<4%)) allowing mean RF values to be applied to all the data. The correlation coefficients (R^2^) of nearly all VOCs were above 0.99 (mean = 0.9954 ± 0.0075%), although there was a slight anomaly in the case of AA (0.9619 (SRD = 0) and 0.9698 (SRD-9)). In addition, to assess reproducibility, the L-WS with a mean of 26.1 ng·μL^−1^ was analyzed repeatedly prior to analysis of each batch of samples (SRD -0, 1, 3, 6, and 9). If the RF values of the 19 VOCs determined using this consecutive series of analyses, the RSE values generally fell below 4% (mean RSE = 1.39 ± 0.82%).

### 2.3. The Analysis of Ammonia and Reduced Sulfur Species

Although strawberries are noted for their attractive fragrance, the fruit can release unpleasant odors if stored for too long. Important offensive odorants such as ammonia and reduced sulfur compounds (RSC) [16,17] were therefore also quantified in this study. As expected, these compounds were found to be difficult to detect in the fresh fruit but they became increasingly abundant as the decay progressed. The TD-GC-MS setup optimized for the VOC analysis in this study is not optimal choice for ammonia or RSCs, thus these compounds were determined using alternative analytical approaches.

For the collection of samples to analyze for ammonia and RSC, the bag sampling method (PEA bag) was employed. The inlet and outlet of the impinger filled with the strawberry samples were connected to a gas cylinder filled with ultra-pure air (>99.999%) and a 10 L empty PEA bag, respectively. Teflon tubing was used to connect the impinger and the gas cylinder at one end and the empty PEA bag at the other. Air for the cylinder was plowed through the impinger containing the strawberry sample and into the 10 L empty PEA bag at a flow rate of 100 mL·min^−1^ for 100 min afterapre-purge was conducted by supplying ultra-pure air into the impinger at a flow rate of 100 mL·min^−1^ for 10 min. Ammonia was analyzed using absorption photometry (Genysys 10 series, Thermo Scientific, Waltham, MA, USA) based on the indophenol method, a well-known approach for amino compounds [18,19]. The reduced sulfur compounds were analyzed using an on-line thermal desorption system (UNITY-Air Server, Markes International, Ltd.) coupled with a GC (CP-3800, Varian, Palo Alto, CA, USA) and pulsed flame photometric detector (PFPD: Varian). The RSCs in the PEA bag were transferred to the TD system using pump and collected into the focusing trap (cold trap) in TD system. The RSCs loaded on the focusing trap were then thermally desorbed and transferred to the GC column for separation and detection. Finally, the dilution-to-threshold (D/T) ratios of the strawberry samples were also determined using an air dilution sensory (ADS) test, as a direct means to assess odor intensity [20].

## 3. Results and Discussion

### 3.1. Major Volatile Components Emitted from Strawberry Samples

The concentration and occurrence frequency of these species is classified in terms of functional groups in Table 1. The types and concentration levels of 147 VOCs detected from all strawberry samples are also summarized in Table 6S. If they are arranged by the chemical grouping and occurrence frequency, they can be classified as follows: (1) ester = 61, (2) alcohol = 21, (3) aldehyde = 11, (4) ketone = 9, (5) fatty acid = 5 and (6) miscellaneous (*etc.*) = 40.

Esters (n = 47), with total concentration of 52,648 μg· m^−3^ (76.8% by mass), represented more than half of the 81 VOCs detected at the start of the study (SRD = 0). Similarly, approximately 130 different types of esters have been reported from strawberry fragrances [21], where they were found to represent 25 to 90% of strawberry volatiles [22-24]. At SRD-1, esters (n = 38) still recorded the highest concentration (42,713 μg· m^−3^). By day 3, while the total concentration of esters had continued to increase (sum concentration = 705,447 μg· m^−3^) their numbers had fallen down to 31. However, once decay had started, their concentration dropped dramatically down to 533 (SRD = 6) and 45.7 μg·m^−3^ (SRD = 9). In contrast, alcohols exhibited a reversed trend. Although alcohols were much less abundant in fresh strawberries (sum concentration of SRD-0, 1, and 3 = 1,582 μg· m^−3^), they tended to peak noticeably at 380 mg·m^−3^ on SRD-6. Thus, the best indicators of the fresh and decayed stages of strawberries are identified as esters and alcohols, respectively. If the other classes of chemicals are considered, aldehydes were detected in all samples and recorded the highest concentration (19,054 μg· m^−3^) on day 3. Ketones and fatty acids were also relatively abundant in fresh strawberries, although they faded away during decay (Figure 2).

In order to evaluate the indicative fragrance of strawberry, the relative composition (RC) of the strawberry volatiles was assessed initially by normalizing the concentration of an individual compound against the total concentration of all species at each sampling day (Table 2). If any compound with more than 0.05% of RC (total mass) on one or more sampling day was selected, 53 were observed. The compound contribution pattern of these 53 VOCs was then analyzed both in terms of concentration and odor intensity. The sum concentration for these major VOCs (RC > 0.05%) generally exceeded 99.9% of the total mass of VOCs from each individual measurement (99.4% (SRD-0) to 99.99% (SRD-6).

Ethyl acetate [mass concentration = 17,240 μg·m^−3^ (25.1%)] and methyl acetate 11,945 μg·m^−3^ (17.4%) were the highest from the SRD-0 sample along with ethyl butyrate (7,290 μg·m^−3^), methyl butyrate (4,977 μg·m^−3^), ethyl hexanoate (4,270 μg·m^−3^), and methyl hexnoate (2,492 μg· m^−3^). These six esters thus showed the highest RC (70.3%) at SRD-0. Other than esters, acetone and acetic acid had relatively high concentrations of 5,960 and 6,177 μg· m^−3^ at SRD-0.

If the results of all fresh stages (SRD-0, 1, and 3) are combined together, esters maintained the maximum abundance (n = 23) with the sum of 80,015 μg· m^−3^ (93.4%). However, patterns changed dramatically during decay, esters dropped down to 20 μg· m^−3^ (n = 22) in SRD-6 after excluding ethyl acetate (SRD-6 = 467 μg·m^−3^). Acetaldehyde also underwent 15-fold reduction to 1,313 μg· m^−3^ in SRD-6 compared to its maximum at (SRD-3). In contrast, two alcohols rose significantly to 287,758 μg·m^−3^ [75.3% (isobutyl alcohol)] and 91,537 μg·m^−3^ [23.9% (ethyl alcohol)] at SRD-6. In the case of SRD-9, the concentrations of those alcohols decreased to 967 (15.9%) and 2,665 μg·m^−3^ (43.8%), respectively. Moreover the sum quantity (μg· m^−3^) of strawberry volatiles detected recorded the lowest value of 6,055 at SRD-9 [(RC [SRD-9/Σ SRD-all] × 100) = 0.49%] compared with all other periods (58,281 (SRD-2) to 730,144 μg· m^−3^ (SRD-3)).

Although our analysis focused mainly on volatile organics by GC-MS, we also analyzed some offensive odorants like reduced sulfur compounds (RSC) and NH_3_ (Table 7S). It can be seen that three RSCs and ammonia were detected from the strawberry sample. Especially, methane thiol and dimethyl disulfide were seen fairly consistently and recorded fairly high concentrations of 267 μg· m^−3^ and 196 μg m^−3^ in SRD-1, respectively. In contrast, ammonia was detected apparently only under the decaying conditions (concentration (μg· m^−3^) = 169 (SRD-6) and 445 (SRD-9)) relative to the fresh period below 81.3 (SRD-0, 1, and 3).

### 3.2. The Variety of VOC Threshold Values for Strawberry Volatiles and Their Relationship with Molecular Weights

The odor threshold of a compound is defined as the lowest concentration that can be detected by human olfaction [25]. The lower the odor threshold, the stronger the odorant will be. However, many authors have investigated the threshold values of various volatiles and results for individual compounds can be very variable. In this study, a literature survey was conducted for the odor strengths (thresholds) of the strawberry volatiles. Although we measured a total of 147 VOCs during this study period, we were only able to obtain threshold values for up to of 79 species (Table 6S). The results of this survey are also summarized in Table 8S.

As reported previously, the odor strengths of VOCs tend to exhibit strong relationships with their physicochemical properties, e.g., the number of carbons and molecular weight [26]. Hence, a number of combinations between such variables (e.g., log thresholds *vs.* molecular weights) were tested to seek for such linear relationship. For this comparative analysis, fatty acids and some miscellaneous groups were however excluded due to the lack of threshold data. As shown in Table 9S, an inverse correlation was seen consistently between the log (thresholds) and molecular weights of VOCs without a single exception. However, the magnitude of slope values differed greatly between the VOC groups, while the strongest correlation with molecular weights was seen from the maximum (out of all available) threshold values.

As shown in Figure 3, the strongest correlations were seen from a pair of log-maximum threshold values and molecular weight among all matching combinations (<1> for all data (n = 62): (1) R^2^ (maximum) = 0.4260, (2) R^2^ (minimum) = 0.2171, and (3) R^2^ (geometric mean) = 0.3384, and <2> For optimal fit (n = 54): (1) R^2^ (maximum) = 0.5743, (2) R^2^ (minimum) = 0.2897, and (3) R^2^ (geometric mean) = 0.4473).

### 3.3. The Evaluation of the Odor Strengths with Changes in Freshness Status

In previous sections, the changes of VOC quantities and their threshold values were evaluated from fresh to decayed stages of strawberry. To learn more about strawberry fragrance, our results were examined further with respect to type and strength of strawberry odors. The selection of reasonable threshold value is important to help understand the contribution of a compound at its given concentration level to the overall perception of odor. It is however difficult to assign a single meaningful figure because the threshold of a given compound is often available as multiple reported values.

In this study, the odor strengths of strawberry were calculated in terms of odor activity values (OAV) by dividing the concentrations of the VOCs with the corresponding threshold in the same concentration unit: OAV = concentration (ppbv)/threshold value (ppbv) [27]. For the 53 selected major VOCs, multiple threshold values have been reported for many (21 (one value), 21 (two values), 10 (three values), and 1 (four values)). In case of two or more reported value thresholds, the one with maximum value was used to calculate the OAVs in light of consistency as seen in correlation analysis.

Table 3 presents the specific description of odor types for each of the major VOCs with their OAV (n = 53). Information of the odor types was obtained by surveying the GC-olfactometry analysis of VOC [refer to a list of references (n = 24) in Table 3]. The ΣOAV values of the strawberries tended to decrease abruptly with storage time from their maximum at SRD-0 (OAV from SRD-0 to SRD-9 were 12,972, 6,992, 2,524, 196 and 7.37, respectively). The OAV values at SRD-0 decreased in order of ethyl butyrate (6,160), ethyl hexanoate (3,608), ethyl isovalerate (1,592), and ethyl 2-methylbutyrate (942).

To assess the relative contribution of a given compound in terms of OAV, its relative proportion (RP) was also calculated by dividing OAV (a given compound) with ΣOAV (all) (Table 3). This RP term for OAV is distinguished from the RC term used for relative mass concentration. If OAVs of these four esters are summed, their RP represents 94.8% of total OAV at SRD-0. It thus suggests that the fragrance of fresh strawberries is governed predominantly by these four esters. The scent of these esters is characterized as fruity, apple, and sweet (Table 3). Although their OAVs decreased from SRD-0 to SRD-1, they still recorded the highest OAVs among all the VOCs evaluated at SRD-1 with ΣRP = 91.1%. The fragrance pattern of SRD-0 and -1 is not likely to have changed because the key volatiles (the four esters) remained constant. In case of SRD-3, esters had high OAV along with significantly large ΣRP (97.2%), although their ΣOAV decreased considerably to 2,453 in SRD-3 relative to the earlier period (SRD-0 (12,917) and SRD-1 (6,942)). As a result, we were able to confirm that the esters should dominate the quality of fresh strawberry fragrance (SRD-0, 1, and 3).

To evaluate the occurrence patterns of fresh strawberry volatiles, our results were compared to a number of previous studies. Du *et al.* [1] analyzed the volatiles emitted from fresh strawberries using solid-phase micro-extraction (SPME)-GC-MS analysis. A total of 54 volatiles from two cultivar samples (Strawberry Festival and Florida Radiance) were selected as the main target compounds (with their respective standards). It is interesting to note that 52 target volatiles were found in both samples, while 23 of them were seen consistently in all fresh stage samples in this study. They further calculated the OAVs of detected volatiles using thresholds of 44 compounds. Accordingly, OAVs were seen to be dominated by two esters (ethyl butyrate and methyl butyrate), 2,5-dimethyl-4-hydroxy-3(2*H*)-furanone (DMHF), and linalool in two samples [(1) Strawberry Festival = 461 (ethyl butyrate: RP = 28.7%), 358 (methyl butyrate: 22.3%), 424 (DMHF: 26.4%), and 102 (linalool: 6.3%) and (2) Florida Radiance = 553 (ethyl butyrate: 33.7%) and 261 (methyl butyrate: 15.9%), 359 (DMHF: 21.9%), and 162 (linalool: 9.9%)]. In this study, ethyl butyrate exhibited relatively high OAV in fresh stages with its RP ((1) 47.5% (SRD-0), 65.4% (SRD-1), and 27.2% (SRD-3)). Likewise, methyl butyrate also showed high OAV with the RP values of 1.30% (SRD-0), 2.79% (SRD-1), and 0.50% (SRD-3). In contrast, the RP values of DMHF and linalool were relatively insignificant, although they were selected as the major VOCs in this study.

Nuzzi *et al.* [12] analyzed strawberry fragrances of six different cultivars such as ApoScaligera ((1) Darselect, (2) Eva, and (3) VR4) and Cesena area in Italy ((4) Alba, (5) Dora, and (6) CS4). The volatiles from these samples were collected by a charcoal adsorption tube and extracted using dichloromethane solution. Lastly, the GC-MS analysis of these extracts yielded a total of 37 volatiles (ester = 28, alcohol = 2, sulfide = 2, lactone = 2, and 3 others (2-pentanone, limonene, and linalool)) in their fresh stage. If the OAV is computed for each compound, nine of them showed the highest contributions with ΣRP = 96.7% (consisting of methyl 2-methylbutyrate (3.46%) to dimethyl trisulfide (23.2%)). Eight out of the nine volatiles with the high RP measured in studies of Nuzzi *et al.* [12] were also detected from our fresh strawberry samples (SRD-0 ,1, and 3). Especially, ethyl butyrate, ethyl 2-methylbutyrate, ethyl hexnoate, and ethyl isovalerate had the highest contribution to OAV in fresh periods. As such, the results of previous studies confirmed the significant role of esters in the fresh strawberries [1,12].

**Figure4:**
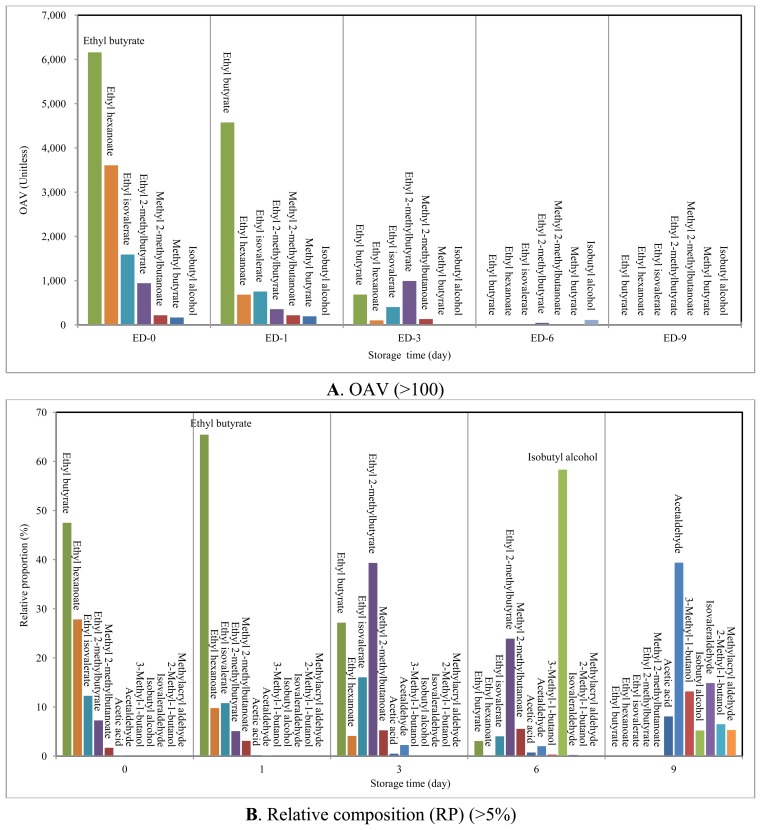
Comparison between (odor activity value) OAV and relative proportion (RP) of the major VOCs which had OAV and RP of above 100 and 5%, respectively at storagetime of 0, 1, 3, 6, and 9 days.

During decay, ΣOAVs of all detected esters (except ethyl 2-methylbutyrate with OAV = 46.9) were below 10, while accounting for 12.9% by ΣRP (ester (n = 12)). In contrast, isobutyl alcohol exhibited the highest OAV of 114 in SRD-6 (RP = 58.3%). As the odor of isobutyl alcohol is characterized as plastic and bad, is is distinguishable from pleasant fresh scents. As a result, odor intensity decreased with the progress of strawberry decay with the emergence of some offensive odors (e.g., ΣOAV of 7.37 in SRD-9). In SRD-9, only two compounds (acetaldehyde and isovaleraldehyde) showed OAVs above 1. For the reader's reference, volatiles that showed up at least once in terms of either OAV (above 100) or RP (above 5%) during the whole study period are illustrated in Figure 4.

If the OAVs of the RSCs and ammonia detected in strawberry aroma profiles are examined, their values from decayed strawberry samples (SRD-6 and 9) generally had very low OAV (below 1). Only methanethiol had high OAVs above 10 in SRD-0 and SRD-1 (OAV = 14.9 (SRD-0) and 64.6 (SRD-1)] compared with other RSCs or ammonia. However, if these results are compared with ΣOAV (all) of all hydrocarbons in fresh strawberry samples (SRD-0 and 1), the RP value of methane thiol were as low as 0.13% (SRD-0) and 1.04% (SRD-1). As a result, it is reasonable to infer that the volatile hydrocarbons should represent the odor/fragrance characteristics of strawberry most effectively.

### 3.4. Comparison between Odor Activity Value (OAV) and Dilution-to-Threshold Ratio

In this section, the TD-GC-MS odor profiles were compared between fresh and decaying strawberries. The results were then evaluated to assess the relationship between the classes of volatile components and their odor intensity. To estimate the key volatiles which dominate the strawberry scent, the odor strengths (OAV) were examined against the relative (mass) composition. Evaluation of our data indicated that the use of ΣOAV was useful to assess the actual occurrence of fragrance/odor from strawberry samples. As another means to explore the odor intensities of strawberry samples, we estimated the dilution-to-threshold (D/T) ratio derived experimentally based on air dilution sensory (ADS) test [20]. The D/T ratio is commonly used as a tool to measure the level of dilution by which the odor threshold is recognized [20]. As the D/T ratio of strawberry samples was measured concurrently with the analysis of their chemical composition, the ΣOAV values at each SRD interval can also be evaluated in relation to the D/T ratio.

The results of correlation analyses between storage day and log odor intensities (log ΣOAV and log D/T ratio) are plotted in Figure S3(A). In compliance with general expectation, the log D/T ratio decreased with the progress of decay to show the slope values of -0.2185 (with R^2^ = 0.8646 and p-value = 2.21E-2) (log D/T ratio). A similar trend is also observable from ΣOAV. As such, the results of this correlation analysis between the log ΣOAV and log D/T ratio show a strong correlation with R^2^ = 0.9338 (p-value = 7.33E-3). As a result, the computation of ΣOAV values can be used as sensitively as that of the D/T ratios to assess the fragrance occurrence pattern. In other words, the ΣOAV should be considered a good criterion to assess the fragrance or odor intensity of strawberry samples.

## 4. Conclusions

The volatiles emitted from strawberry are important components to accurately assess its fragrance. Although the fragrance of the strawberry can influence its flavor and taste, its smell types can vary greatly with aging conditions. If the volatiles from strawberry were analyzed at the fresh stage, the results should be useful enough to understand the natural flavor of strawberries. In this study, the mass concentrations and odor strength (odor activity values: OAV) from strawberries were analyzed by the sorbent tube method at storage times of 0, 1, 3, 6 and 9 days at 25 °C.

The results of our analysis indicated that the mass concentration (μg·m^−3^) of all strawberry volatiles varied greatly over time: 68,569 (SRD-0), 58,503 (SRD-1), 730,593 (SRD-3), 382,245 (SRD-6), and 6,086 (SRD-9). The concentrations of strawberry volatiles released at fresh period before (SRD-3) were absolutely dominated in this order, ethyl acetate, methyl acetate, ethyl butyrate, methyl butyrate, acetaldehyde, acetic acid, and acetone (ΣRC of 97.4%). However, as the strawberry samples began to decay, its RC was dominated by two alcohols (isobutyl alcohol and ethyl alcohol) with significant reductions in their summed concentration.

In this study, we were able to quantify a total of 147 strawberry volatiles (with 79 corresponding odor threshold values from literature survey). In order to assess the fragrance/odorant characteristics of strawberries, the OAV values were calculated for a total of 53 major volatiles that comprised more than 0.05% in mass concentration of all strawberry volatiles. The OAV values decreased abruptly with storage time (ΣOAV: SRD-0 (12,972) to SRD-9 (7.37)). If the ΣOAV values of a given strawberry volatiles are computed for the whole fresh period, their magnitude was dominated by four esters with fruity and sweet scents [(1) ethyl butyrate (11,422), (2) ethyl hexanoate (4,395), (3) ethyl isovalerate (2,751), and (4) ethyl 2-methylbutyrate (2,290)] [ΣRP (four esters) of 92.8%]. However, relative dominance of esters as the key strawberry fragrance changed abruptly with the onset of decay. Hence, as the transition proceeds, relationships between key parameters tended to vary widely. In case of SRD-6, the ΣOAV of the four esters decreased to 60.9 with ΣRC of below 1%, while isobutyl alcohol exhibited the highest OAV of 114 with RC of 75.3%. The ΣOAV was reduced further to reach the minimum value of 7.37 in SRD-9.

In this study, the strawberry volatiles were analyzed from fresh stage to 9 day storage at 25 °C, and the concentration of the strawberry volatiles were evaluated in relation to their occurrence patterns and olfaction data derived as D/T ratio. The overall results of our study suggest that strawberry volatiles are useful indicators to characterize the flavor changes of strawberry at the latter stages of its storage period.

## Figures and Tables

**Figure 1.**
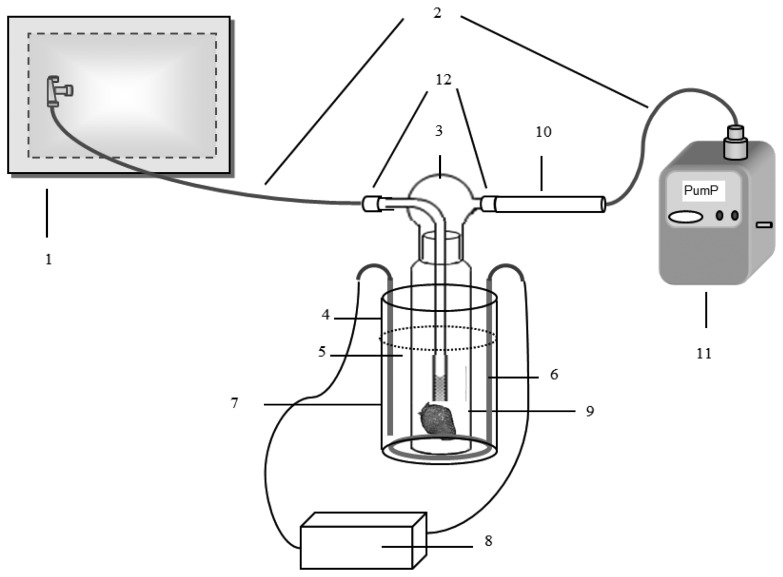
Illustration of the impinger system for the collection of VOCs emitted from strawberry samples. Labels: (**1**) 10 L polyester aluminum bag filled with ultra-pure nitrogen; (**2**) Silicon tubing; (**3**) Impinger bottle; (**4**) Aluminum container; (**5**) Water heated to 25 °C; (**6**) Heater; (**7**) Sensor; (**8**) Temperature regulator; (**9**) Strawberry slices; (**10**) Sorbent tube; (**11**) Mini vacuum pump; and (**12**) Teflon tubing.

**Figure 2.**
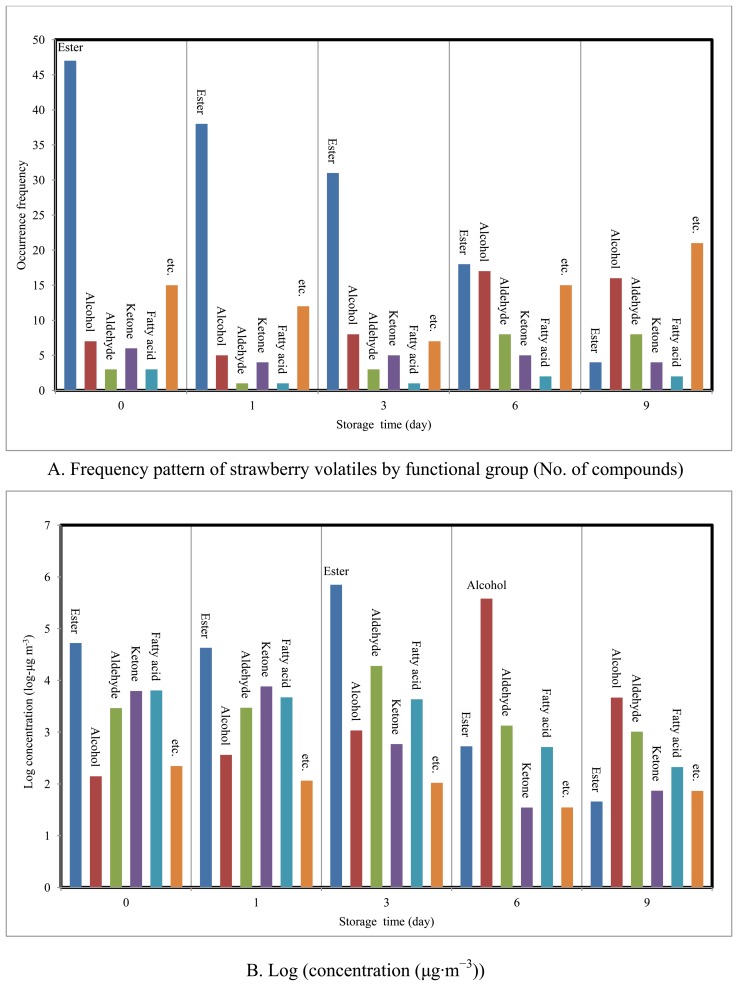
Comparison of frequency) pattern and log concentration of strawberry volatiles (compounds sorted by functional group).

**Figure 3.**
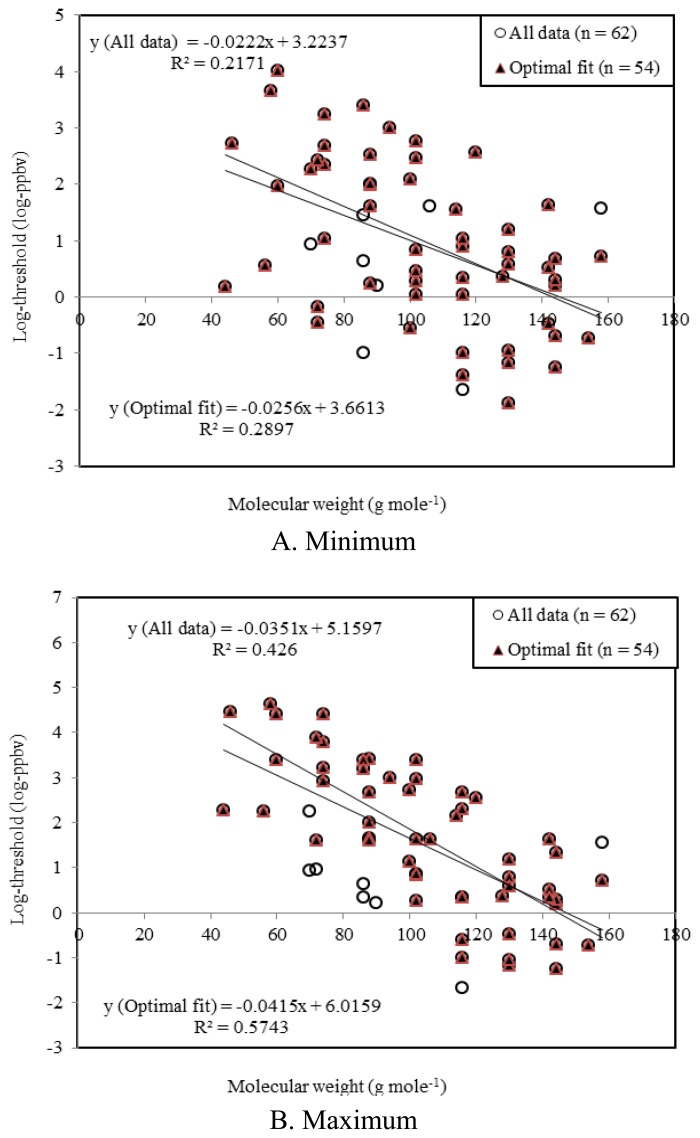

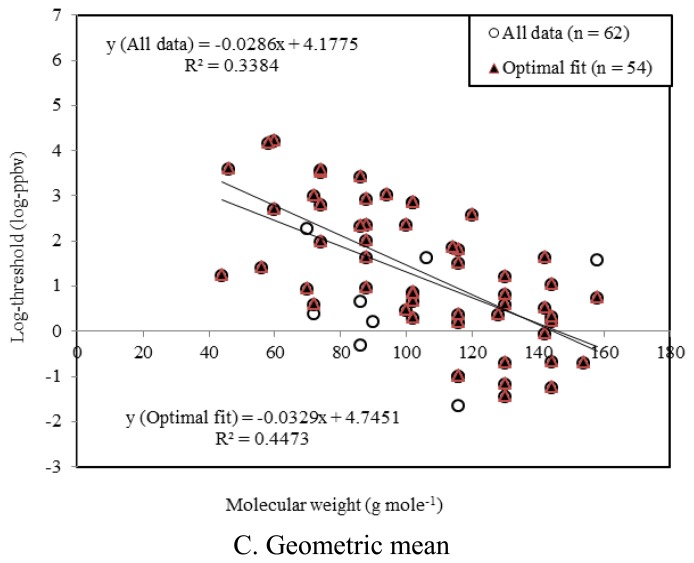
Plots of correlation between molecular weight and log (odor thresholds (ppbv)) for alldata (n = 62) and optimal fit (n = 54) of the four major VOC groups (Ester, Alcohol, Aldehyde, and Ketone) emitted from strawberry samples.

**Table 1.** Occurrence frequency and summed concentration of VOC groups emitted from strawberry samples during the whole study period.

**Sample Code**	**Functional Group**						**Total**

	**A. Ester**	**B. Alcohol**	**C. Aldehyde**	**D. Ketone**	**E. Fatty Acid**	**F.*etc.***	
[A] Frequency (Number of chemical compounds for each functional group)							
SRD-0	47	7	3	6	3	15	81
SRD-1	38 (34) a	5 (4)	1 (1)	4 (4)	1 (1)	12 (7)	61(51)
SRD-3	31 (24)	8 (3)	3 (1)	5 (2)	1 (1)	7 (5)	55 (36)
SRD-6	18 (14)	17 (7)	8 (2)	5 (2)	2 (1)	15 (3)	65 (29)
SRD-9	4 (3)	16 (16)	8 (7)	4 (4)	2 (1)	21 (8)	55 (39)

Total	61	21	11	9	5	40	147

[B] Summed concentration (ug m^−3^)							
SRD-0	52,648	140	2,918	6,251	6,390	221	68,569
SRD-1	42,713	364	2,959	7,649	4,703	115	58,503
SRD-3	705,447	1,078	19,054	589	4,319	105	730,593
SRD-6	533	379,789	1,338	35.0	516	35.1	382,245
SRD-9	45.7	4,658	1,022	73.9	212	73.4	6,086

Total	801,386	386,029	27,292	14,598	16,139	550	1,245,995

a No. of compounds detected consistently from the previous sample is given in the parenthesis: 33 out of 37 compounds in SRD-1 were for example, also seen in SRD-0.

**Table 2.** Concentration (in both ppb and μg· m^−3^) and the corresponding relative (mass) composition (RC) of the major strawberry volatiles (mass concentration abundance a ≥ 0.05 %: n = 53).

Order	Compounds	Concentration										Relative composition a (RC: %)				
	
		ppb					μgm^−3^									
		
		SRD-0	SRD-1	SRD-3	SRD-6	SRD-9	SRD-0	SRD-1	SRD-3	SRD-6	SRD-9	SRD-0	SRD-1	SRD-3	SRD-6	SRD-9
**A. Ester**																
1	Methyl acetate	3,949	3,875	71,077	1.57	0.75	11,945	11,720	214,987	4.76	2.28	17.4	20.0	29.4	1.25E-03	0.04
2	Ethyl acetate	4,793	4,277	134,969	130	11.8	17,240	15,384	485,476	467	42.5	25.1	26.3	66.4	0.12	0.70
3	Methyl propionate	40.8	51.0	23.6	0.17	8.75E-03	147	183	85.0	0.63	3.15E-02	0.21	0.31	0.01	1.64E-04	
4	S-Methyl thioacetate	94.2	129.3	3.07E-02	3.07E-02	3.07E-02	346	476	1.13E-01	1.13E-01	1.13E-01	0.51	0.81			
5	Isopropyl acetate	52.8	16.7	27.5	4.95E-03	4.95E-03	220	69.5	114	2.06E-02	2.06E-02	0.32	0.12	0.02		
6	Ethyl propionate	52.7	5.19E-03	53.5	5.19E-03	5.19E-03	220	2.17E-02	223	2.17E-02	2.17E-02	0.32		0.03		
7	Propyl acetate	5.19E-03	5.19E-03	185	0.72	5.19E-03	2.17E-02	2.17E-02	769	3.02	2.17E-02			0.11	7.90E-04	
8	Methyl butyrate	1,194	1,384	88.8	0.47	5.19E-03	4,977	5,772	370	1.95	2.17E-02	7.26	9.9	0.05	5.10E-04	
9	Methyl 2-methylbutanoate	21.8	21.7	13.2	1.08	3.35E-03	103	103	62.7	5.14	1.59E-02	0.15	0.18	0.01	1.35E-03	
10	Isobutyl acetate	9.11	7.00	88.9	1.86	3.35E-03	43.2	33.2	421	8.80	1.59E-02	0.06	0.06	0.06	2.30E-03	
11	Methyl isovalerate	54.8	56.9	7.78	3.35E-03	3.35E-03	260	270	36.9	1.59E-02	1.59E-02	0.38	0.46	0.01		
12	Ethyl butyrate	1,537	1,142	171	1.50	3.48E-03	7,290	5,415	812	7.10	1.65E-02	10.6	9.3	0.11	1.86E-03	
13	Butyl acetate	34.2	36.5	26.4	0.05	3.49E-03	162	173	125	0.24	1.65E-02	0.24	0.30	0.02	6.19E-05	
14	Isopropyl butyrate	30.7	25.9	2.43E-03	2.43E-03	2.43E-03	163	138	1.29E-02	1.29E-02	1.29E-02	0.24	0.24			
15	Ethyl 2-methylbutyrate	63.0	23.8	66.3	3.13	2.43E-03	335	126	352	16.6	1.29E-02	0.49	0.22	0.05	4.35E-03	
16	Ethyl isovalerate	142	67.2	36.1	0.70	2.43E-03	753	357	192	3.73	1.29E-02	1.10	0.61	0.03	9.76E-04	
17	Isoamyl acetate	81.3	69.1	165	1.21	2.43E-03	432	367	878	6.43	1.29E-02	0.63	0.63	0.12	1.68E-03	
18	Ethyl valerate	7.31	1.73	2.51E-03	2.51E-03	2.51E-03	38.8	9.22	1.33E-02	1.33E-02	1.33E-02	0.06	0.02			
19	Methyl hexanoate	469	185	11.4	2.51E-03	2.51E-03	2,492	982	60.3	1.33E-02	1.33E-02	3.63	1.68	0.01		
20	trans-2-Hexenyl acetate	38.6	1.78	1.90E-03	1.90E-03	1.90E-03	224	10.4	1.11E-02	1.11E-02	1.11E-02	0.33	0.02			
21	Ethyl hexanoate	725	137	20.8	0.03	1.90E-03	4,270	808	123	0.16	1.12E-02	6.23	1.38	0.02	4.28E-05	
22	Hexyl acetate	110	25.5	4.70	1.90E-03	1.90E-03	647	150	27.7	1.12E-02	1.12E-02	0.94	0.26	3.79E-03		
23	Methyl octanoate	5.84	0.88	1.49E-03	1.49E-03	1.49E-03	37.7	5.71	9.64E-03	9.64E-03	9.64E-03	0.06				

	ΣConcentration or its RC (ester)						52,346	42,553	705,116	526	44.7	76.3	72.7	96.5	0.14	0.74

**B. Alcohol**																
1	Ethyl alcohol	3.72E-02	3.72E-02	309	48,685	1,417	7.00E-02	7.00E-02	580	91,537	2,665			0.08	23.9	43.8
2	Isopropyl Alcohol	8.76	4.58	45.3	92.8	278	21.5	11.2	111	228	681	0.03	0.02	0.02	0.06	11.2
3	n-Propyl alcohol	1.42E-02	1.42E-02	30.4	46.6	36.1	3.49E-02	3.49E-02	74.5	114	88.5			0.01	0.03	1.45
4	Isobutyl alcohol	7.01E-03	7.01E-03	31.8	95,136	320	2.12E-02	2.12E-02	96.3	287,758	967			0.01	75.3	15.9
5	Isopropenylethyl alcohol	4.86E-03	4.86E-03	4.86E-03	2.41	4.47	1.71E-02	1.71E-02	1.71E-02	8.48	15.7				2.22E-03	0.26
6	3-Methyl-1-butanol	1.09	1.28	50.7	26.9	43.3	3.91	4.61	182	96.9	156	0.01	0.01	0.02	0.03	2.56
7	2-Methyl-1-butanol	4.65E-03	4.65E-03	4.65E-03	9.48	19.6	1.67E-02	1.67E-02	1.67E-02	34.1	70.5				0.01	1.16
8	n-Pentanol	4.84E-03	4.84E-03	4.84E-03	0.99	0.86	1.74E-02	1.74E-02	1.74E-02	3.57	3.10				9.33E-04	0.05
9	1-Chloro-2-propanol	20.0	80.3	8.91E-03	8.91E-03	8.91E-03	77.0	309	3.42E-02	3.42E-02	3.42E-02	0.11	0.53			
10	n-Hexanol	0.52	3.34E-03	1.16	0.15	1.08	2.17	1.39E-02	4.83	0.63	4.51	3.16E-03		6.61E-04	1.65E-04	0.07
11	Linalool	5.14	5.08	1.17E-03	1.17E-03	1.17E-03	32.4	32.0	7.34E-03	7.34E-03	7.34E-03	0.05	0.05			

	ΣConcentration or its RC (alcohol)						137	356	1,049	379,781	4,651	0.20	0.61	0.14	99.4	76.4

**C. Aldehyde**																
1	Acetaldehyde	1,618	1,646	10,584	730	540	2,909	2,959	19,035	1313	971	4.24	5.06	2.61	0.34	16.0
2	Allyl aldehyde (Acrolein)	8.24E-02	8.24E-02	8.24E-02	5.08	6.90	1.89E-01	1.89E-01	1.89E-01	11.6	15.8				3.04E-03	0.26
3	Methylacryl aldehyde	1.59E-02	1.59E-02	1.59E-02	1.80	3.33	4.54E-02	4.54E-02	4.54E-02	5.14	9.52				1.34E-03	0.16
4	Isobutyraldehyde	1.46E-02	1.46E-02	1.46E-02	0.24	3.80	4.30E-02	4.30E-02	4.30E-02	0.71	11.2				1.85E-04	0.18
5	Isovaleraldehyde	8.52E-03	8.52E-03	8.52E-03	1.03	2.45	2.99E-02	2.99E-02	2.99E-02	3.60	8.62				9.43E-04	0.14

	ΣConcentration or its RC (aldehyde)						2,909	2,959	19,035	1,334	1,016	4.24	5.06	2.61	0.35	16.7

**D. Ketone**																
1	Acetone	2,514	3,131	224	7.35	28.0	5,960	7,422	532	17.4	66.5	8.69	12.7	0.07	4.56E-03	1.09
2	***Methyl ethyl ketone***	1.38E-02	1.38E-02	1.38E-02	1.15	1.35	4.05E-02	4.05E-02	4.05E-02	3.39	3.96				8.86E-04	0.07
3	Methyl n-propyl ketone	54.0	47.1	7.55E-03	7.55E-03	7.55E-03	190	165	2.66E-02	2.66E-02	2.66E-02	0.28	0.28			
4	***Methyl Isobutyl Ketone***	4.62	7.50	1.11	4.11E-03	4.11E-03	18.9	30.7	4.53	1.68E-02	1.68E-02	0.03	0.05	6.19E-04		
5	Methyl amyl ketone	5.64	6.52	3.23E-03	0.51	0.61	26.3	30.4	1.50E-02	2.36	2.85	0.04	0.05		6.18E-04	0.05

	ΣConcentration or its RC (ketone)						6,195	7,649	536	23.2	73.3	9.04	13.1	0.07	6.06E-03	1.20

**E. Fatty acid**																
1	Acetic acid	2,519	1,918	1,761	210	86.3	6,177	4,703	4,319	514	212	9.01	8.04	0.59	1.35E-01	3.48
2	2-PROPYNOIC ACID	69.6	2.35E-02	2.35E-02	2.35E-02	2.35E-02	199.3	6.71E-02	6.71E-02	6.71E-02	6.71E-02	0.29				

	ΣConcentration or its RC (fatty acid)						6,376	4,703	4,319	514	212	9.30	8.04	0.59	1.35E-01	3.48

**E. *etc.***																
1	n-Pentane	12.1	6.63E-03	6.63E-03	0.29	2.20	35.7	1.95E-02	1.95E-02	0.86	6.48	0.05			2.26E-04	0.11
2	Ethyl ether	7.28E-03	7.28E-03	0.70	0.41	1.45	2.20E-02	2.20E-02	2.12	1.23	4.38			2.90E-04	3.21E-04	0.07
3	1,3-Hexadiene	25.0	1.77	4.55E-03	4.55E-03	4.55E-03	83.9	5.94	1.53E-02	1.53E-02	1.53E-02	0.12	0.01			
4	n-Hexane	1.21	1.02	1.02	4.33E-03	8.03	4.27	3.60	3.57	1.52E-02	28.2	0.01	0.01	4.89E-04		0.46
5	Toluene	3.14E-03	3.14E-03	3.14E-03	3.14E-03	2.48	1.18E-02	1.18E-02	1.18E-02	1.18E-02	9.32					0.15
6	Styrene	10.0	12.0	19.4	3.03	1.09	42.7	51.0	82.5	12.9	4.64	0.06	0.09	0.01	3.36E-03	0.08
7	***2,5-Dimethyl-4-methoxy-3(2H)-furanone***	1.42	1.96E-03	1.96E-03	2.66	0.88	8.23	1.14E-02	1.14E-02	15.5	5.08	0.01			4.04E-03	0.08

	ΣConcentration or its RC (etc)						175	60.5	88.2	30.4	58.1	0.25	0.10	0.01	0.01	0.96

	ΣConcentration or its RC (all)						68,139	58,281	730,144	382,209	6,055	99.4	99.6	99.9	99.99	99.5

a RC = [Mass concentration (‘i’th compound) / mass concentration (sum) at a given exp day] s100

b Values below detection limit (BDL) are underlined (calculated as method detection limit)

**Table 3.** Relationship between relative composition (RP) and odor activity values (OAV) of the major VOCs (n = 53) emitted from strawberry.

Order	Compounds	OAV (concentration/ threshold)					Relative proportion * (RP, %)					Odor type / descriptor
	
		SRD-0	SRD-1	SRD-3	SRD-6	SRD-9	SRD-0	SRD-1	SRD-3	SRD-6	SRD-9	
**A. Ester**												
1	Methyl acetate	0.64	0.63	11.52	2.55E-04	1.22E-04	4.93E-03	8.98E-03	0.46	1.30E-04	1.66E-03	
2	Ethyl acetate	1.82	1.63	51.3	0.05	4.49E-03	0.01	0.02	2.03	0.03	0.06	sweet fruit^a^, grape^a^, contact glue^i^, fruity^l^, pinapple^l^
3	Methyl propionate	0.42	0.52	0.24	1.78E-03		3.21E-03	0.01	0.01	9.08E-04		
4	S-Methyl thioacetate	58.6	80.4				0.45	1.15				sulfurous^a^, cheesy^a^
5	Isopropyl acetate	0.02	0.01	0.01			1.70E-04	9.93E-05	4.53E-04			
6	Ethyl propionate	7.53		7.65			0.06		0.30			
7	Propyl acetate			0.19	7.54E-04				0.01	3.85E-04		
8	Methyl butyrate	168	195	12.5	0.07		1.30	2.79	0.50	0.03		apple^a^, fruity^b^, ^c^, ^d^, ^i^, cheese^i^
9	Methyl 2-methylbutanoate	218	217	132	10.86		1.68	3.10	5.25	5.54		green apple^a^, fruity^a^, sweet^a^
10	Isobutyl acetate	0.02	0.01	0.19	3.88E-03		1.47E-04	2.09E-04	0.01	1.98E-03		strawberry^e^, light fruity^i^, flowery^i^
11	Methyl isovalerate	24.9	25.9	3.54			0.19	0.37	0.14			fruity^a^, apple^a^, pineapple^a^
12	Ethyl butyrate	6,160	4,576	686	6.00		47.5	65.4	27.2	3.06		fruity^a^, ^b^, ^c^, ^d^, ^e^, ^i^, sweet^a^, ^b^, ^i^, pineapple^a^, ester-like^b^, strawberry^e^, cheese^i^, fruity sweet^m^, ^p^, ^r^, ^u^
13	Butyl acetate	0.18	0.19	0.14	2.56E-04		1.35E-03	2.68E-03	0.01	1.31E-04		fruity^a^, banana^a^, apple^i^, glue^i^
14	Isopropyl butyrate	4.95	4.18				0.04	0.06				fruity^a^, sweet^a^, pineapple^a^
15	Ethyl 2-methylbutyrate	942	356	993	46.9		7.26	5.08	39.3	23.9		sour^a^, cheesy^a^, sweaty^a^, fruity^d^
16	Ethyl isovalerate	1,592	755	405	7.88		12.3	10.8	16.0	4.02		fruity^a^, ^d^, apple^a^, pineapple^a^, sweet fruit^e^
17	Isoamyl acetate	21.4	18.2	43.5	0.32		0.16	0.26	1.72	0.16		
18	Ethyl valerate	21.9	5.19				0.17	0.07				
19	Methyl hexanoate	30.1	11.9	0.73			0.23	0.17	0.03			fruity^a^, ^b^, ^i^, pineapple^a^, ester-like^b^
20	trans-2-Hexenyl acetate	0.90	0.04				0.01	0.001				
21	Ethyl hexanoate	3,608	683	103.7	0.14		27.8	9.77	4.11	0.07		fruity^a^, ^b^, ^i^, sweet^a^, pineapple^a^, ester-like^b^, green apple^b^, fruit gum^i^
22	Hexyl acetate	54.7	12.7	2.34			0.42	0.18	0.09			fruity^a^, ^i^, green apple^a^, banana^a^, ^i^, apple^i^, pear^i^
23	Methyl octanoate	0.16	0.02				1.23E-03	3.45E-04				Sweet fruity^e^
	
	ΣConcentration (ester)	12,917	6,942	2,453	72.2	4.61E-03	99.6	99.3	97.2	36.8	0.06	

**B. Alcohol**												
1	Ethyl alcohol			0.01	1.69	0.05			4.25E-04	0.86	0.67	
2	Isopropyl Alcohol	3.37E-04	1.76E-04	1.74E-03	3.57E-03	0.01	2.60E-06	2.52E-06	6.90E-05	1.82E-03	0.14	fruity^l^
3	n-Propyl alcohol			0.01	0.02	0.02			5.02E-04	0.01	0.20	Sweet (candy)^l^
4	Isobutyl alcohol			0.04	114	0.38			1.52E-03	58.3	5.22	Plastic^l^, bad^l^
5	Isopropenylethyl alcohol				9.64E-04	1.79E-03				4.92E-04	0.02	
6	3-Methyl-1-butanol	0.02	0.03	1.13	0.60	0.97	1.87E-04	4.10E-04	0.04	0.31	13.2	bitter^e^, harsh^e^, ^k^, Chemical^k^, stale^k^, Alcoholic^r^, green^r^, Fusel oil^s^, pomace^s^
7	2-Methyl-1-butanol				0.23	0.48				0.12	6.49	Alcoholic^s^, green^s^, Fusel oil^t^, pomace^t^
8	n-Pentanol				2.12E-03	1.84E-03				0.001	0.02	
9	1-Chloro-2-propanol	0.02	0.08				1.54E-04	1.15E-03				
10	n-Hexanol	0.01		0.03	3.45E-03	0.02	9.16E-05		1.05E-03	1.76E-03	0.34	winey-fruity^f^, Green^i^, ^k^, ^r^, heavy^i^, nuts^i^, grass^k^, fresh^k^
11	Linalool	27.4	27.0				0.21	0.39				citrus^a^, fruity^a^, ^e^, floral^a^, flowery^b^, ^k^, sweet^b^, lemon^c^, floral-lavender^f^, rose^k^
	
	ΣConcentration or its RC (alcohol)	27.4	27.1	1.22	117	1.94	0.21	0.39	0.05	59.6	26.3	

**C. Aldehyde**												
1	Acetaldehyde	8.70	8.85	56.9	3.92	2.90	6.70E-02	0.13	2.25	2.00	39.4	green apple^a^, fruity^j^, pungent^j^, chemical^k^, alcohol^k^, aldehyde^k^, acetaldehyde^k^, pungent^k^, ^v^, green^n^, ^o^, ^t^, sweet^v^
2	Allyl aldehyde (Acrolein)				0.03	0.04				1.49E-02	0.54	
3	Methylacryl aldehyde				0.21	0.39				0.11	5.31	
4	Isobutyraldehyde				0.01	0.09				3.01E-03	1.27	green^l^, malty^l^, floral^t^
5	Isovaleraldehyde				0.46	1.09				0.23	14.9	green^n^, ^o^, malty^n^, ^o^
	ΣConcentration or its RC (aldehyde)	8.70	8.85	56.9	4.63	4.52	6.70E-02	0.13	2.25	2.36	61.4	
**D. Ketone**												
1	Acetone	0.06	0.07	5.34E-03	1.75E-04	6.68E-04	4.61E-04	1.07E-03	2.12E-04	8.93E-05	9.06E-03	aldehydic^g^, wood pulp^l^, hay^l^
2	***Methyl ethyl ketone***				1.48E-04	1.73E-04				7.56E-05	2.35E-03	Butter^h^, sweet^h^, chocolate^h^, Butterscotch^l^
3	Methyl n-propyl ketone	0.03	0.03				2.69E-04	4.34E-04				thinner^b^, acetone^b^, kerosene^i^, solvents^i^, orange peel^q^, sweet^w^, ^x^, fruity^w^, ^x^
4	***Methyl Isobutyl Ketone***	0.01	0.01	2.06E-03			6.63E-05	2.00E-04	8.17E-05			
5	Methyl amyl ketone	0.04	0.05		3.60E-03	4.34E-03	3.08E-04	6.61E-04		1.83E-03	0.06	meaty^b^, danish blue cheese^i^, green^t^, Animals^r^, blue^r^, cheese^r^
	ΣConcentration or its RC (ketone)	0.143	0.165	7.40E-03	3.92E-03	5.18E-03	1.10E-03	2.36E-03	2.93E-04	2.00E-03	0.07	
**E. Fatty acid**												
1	Acetic acid	17.37	13.23	12.15	1.45	0.60	1.34E-01	0.19	0.48	0.74	8.08	sour^d^, vinager^e^, ^i^, ^k^
2	2-PROPYNOIC ACID	0.74					5.71E-03					
	ΣConcentration or its RC (fatty acid)	18.1	13.2	12.1	1.45	0.60	0.14	0.19	0.48	0.74	8.08	
**E.*etc.***												
1	n-Pentane	3.84E-04			9.29E-06	6.97E-05	2.96E-06			4.74E-06	9.46E-04	
2	Ethyl ether			2.12E-03	1.23E-03	4.39E-03			8.40E-05	6.26E-04	0.06	
3	1,3-Hexadiene	0.01	8.86E-04				9.65E-05	1.27E-05				
4	n-Hexane	5.55E-05	4.67E-05	4.64E-05		3.67E-04	4.28E-07	6.68E-07	1.84E-06		0.005	
5	***Toluene***					1.60E-03					0.02	
6	***Styrene***	0.29	0.34	0.55	0.09	0.03	2.21E-03	0.00	0.02	0.04	0.42	
	2,5-Dimethyl-4-methoxy-3(2H)-furanone											
	ΣConcentration or its RC (etc)	0.73	0.34	0.56	0.90	0.31	0.01	0.00	0.02	0.46	4.15	
	ΣConcentration or its RC (all)	12,972	6,992	2,524	196	7.37	100	100	100	100	100	

* RP = [OAV (n) / OAV (sum of main VOCs) at the exp day] x 100

References:

a Du *et al.* [1],

b Ulrich *et al.* [2],

c Schulbach *et al.* [28],

d Schieberle and Hofmann [29],

e Aznar *et al.* [30],

f Buchbauer *et al.* [31],

g Cai *et al.* [32],

h Clausen *et al.* [33],

i Larsen and Poll [34],

j Semmelroch and Grosch (1995) [35],

k Komes *et al.* [36],

l Arora *et al.* [37],

m Carpino and Mallia [38],

n Kubck ova and Grosch [39],

o Kubícková and Grosch [40],

p Le Quéré *et al.* [41],

q Moio and Addeo [42],

r Moio *et al.* [43],

s Moio *et al.* [44],

t Rychlik and Bosset [45],

u Christensen and Reineccius [46],

v Milo and Reineccius [47],

w Preininger and Grosch [48],

x Preininger *et al.* [49]

**Figure 1S:**
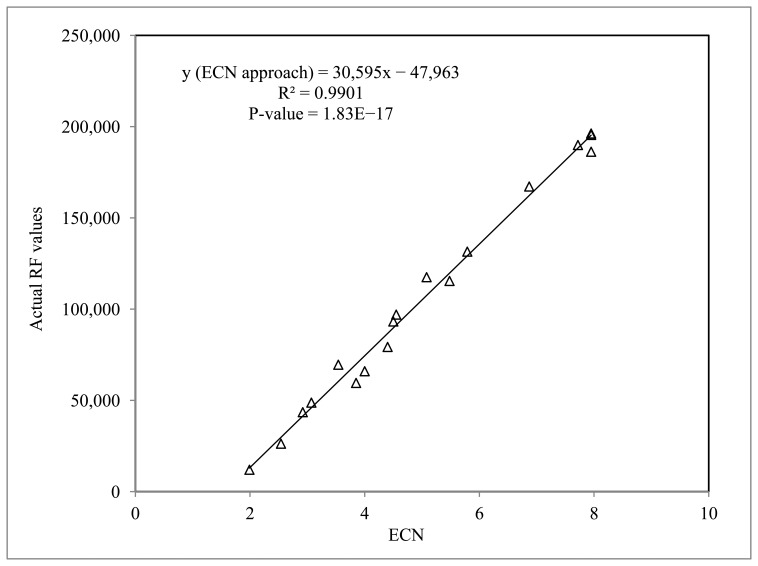
The linear regression analysis between the response factors (RF) and effective carbon number (ECN) for each atom and function group (e.g., C, H, O, >C=O, -O-, and -CH_3_).

**Figure 2S:**
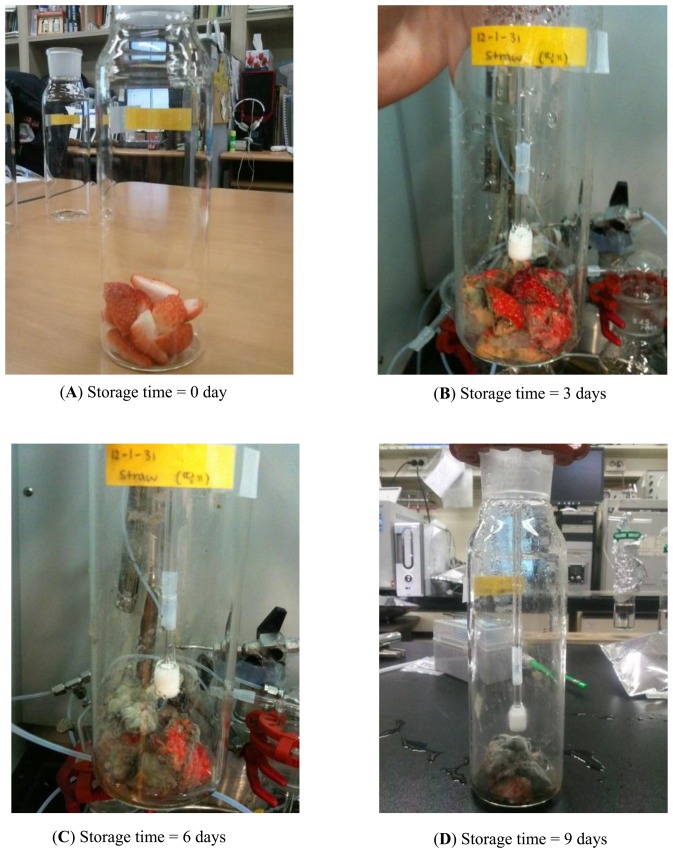
Photographs showing strawberry samples in impinge.

**Figure 3S:**
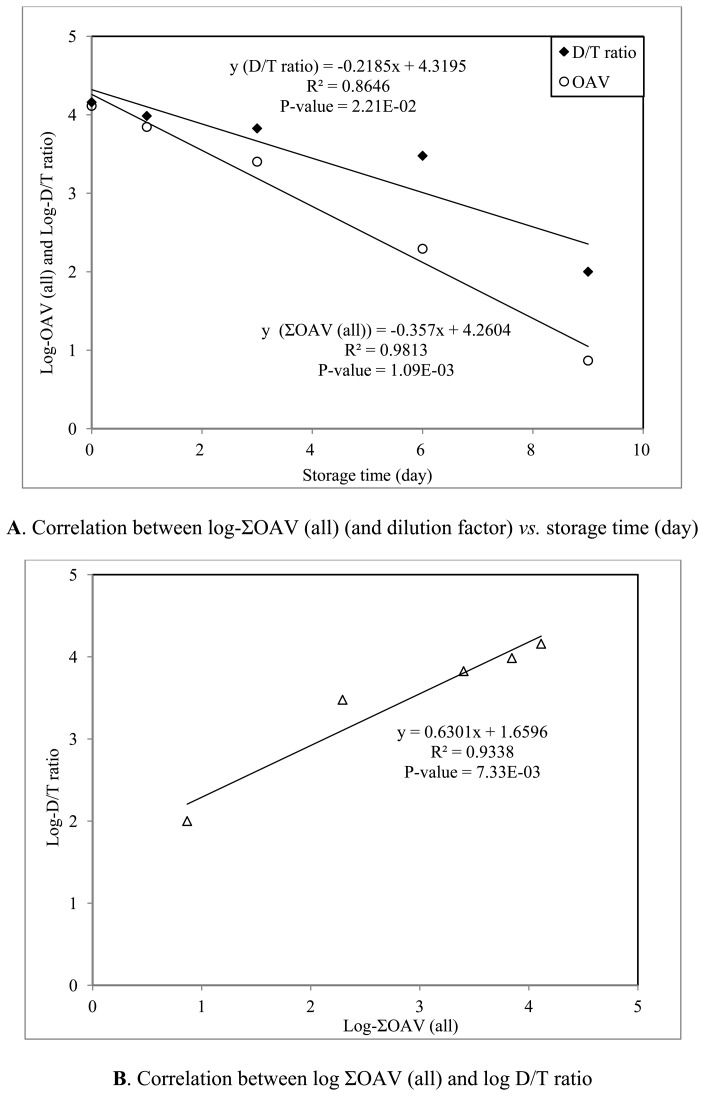
Relationship between Σodor activity values (OAV) and dilution-to-threshold (D/T) ratio.

## Appendix

**Table 1S:** List of 19 VOCs selected as model compounds for the predictive estimation of concentration values for “compounds lacking authentic standards/surrogates (CLASS)” that are emitted from strawberry.

**Order**	**Group**	**Compounds**	**Short name**	**MW (g·mol^−1^)**	**Density (g·cm^−3^)**	**Boiling point (°C)**	**Formula**	**CAS number**
1	Aldehyde	Acetaldehyde	AA	44.1	0.785	20.2	C_2_H_4_O	75-07-0
2		Propionaldehyde	PA	58.1	0.798	46–50	C_3_H_6_O	123-38-6
3		Butyraldehyde	BA	72.1	0.805	74.8	C_4_H_8_O	123-72-8
4		Isovaleraldehyde	IA	86.1	0.797	90–93	C_5_H_10_O	590-86-3
5		n-Valeraldehyde	VA	86.1	0.81	102–103	C_5_H_10_O	110-62-3
	
6	Aromatic	Benzene	B	78.11	0.878	80.1	C_6_H_6_	71-43-2
7		Toluene	T	92.14	0.866	111	C_7_H_8_	108-88-3
8		Styrene	S	104.2	0.906	145	C_8_H_8_	100-42-5
9		*p*-Xylene	p-X	106.2	0.865	138	C_8_H_10_	106-42-3
10		*m*-Xylene	m-X	106.2	0.865	139	C_8_H_10_	108-38-3
11		*o*-Xylene	o-X	106.2	0.88	144	C_8_H_10_	95-47-6
	
12	Ketone	Methyl ethyl ketone	MEK	72.11	0.805	79.64	C_4_H_8_O	78-93-3
13		Methyl isobutyl ketone	MIBK	100.2	0.802	117–118	C_6_H_12_O	108-10-1
	
14	Alcohol	Isobutyl alcohol	i-BuAl	74.12	0.801	108	C_4_H_10_O	78-83-1
	
15	Ester	n-Butyl acetate	BuAc	116.2	0.881	126	C_6_H_12_O_2_	123-86-4
	
16	Fatty acid	Propionic acid	PPA	74.1	0.99	141	C_3_H_6_O_2_	79-09-04
17		*n*-Butyric acid	BTA	88.1	0.958	163.5	C_4_H_8_O_2_	107-92-6
18		*i*-Valeric acid	IVA	102	0.925	175–177	C5H10O2	503-74-2
19		*n*-Valeric acid	VLA	102	0.938	186–187	C5H10O2	109-52-4

**Table 2S:** Preparation of liquid phase VOC standard for the analysis by the TD-GC-MS system.

A. Preparation of liquid phase standard for 19 VOCs																					

**Compound^s^**		**Methanol**	**AA**	**PA**	**BA**	**IA**	**VA**	**B**	**T**	**S**	**p-X**	**m-X**	**o-X**	**MEK**	**MIBK**	**i-BuAl**	**BuAc**	**PPA**	**BTA**	**IVA**	**VLA**
**Primary grade chemical**	Concentration (%)		99.0	97.0	99.0	97.0	97.0	99.5	99.5	99.0	99.0	99.0	97.0	99.0	99.5	99.0	99.5	99.0	99.0	99.0	99.0

**PS^a^**	Volume (μL)	13,700	900	300	300	300	300	300	300	300	300	300	300	300	300	300	300	300	300	300	300
	Concentration (ng·μL^−1^)		34,972	11,611	11,954	11,596	11,786	13,104	12,925	13,454	12,845	12,845	12,804	11,954	11,970	11,895	13,149	14,702	14,226	13,736	13,929

**1st L-WS^b^**	volume (μL)	19,800									200 (of PS)										
			
	Concentration ( ng·μL^−1^)		350	116	120	116	118	131	129	135	128	128	128	120	120	119	131	147	142	137	139

B. Preparation of final working standard (F-WS) for 5 point calibration: absolute mass (ng) of VOC loaded on tube sampler																					

**Order**	**Mixing volume (μL)**		**Concentration c (ng μL^−1^)**																		
	
	**1st L-WS**	**Methanol**	**AA**	**PA**	**BA**	**IA**	**VA**	**B**	**T**	**S**	**p-X**	**m-X**	**o-X**	**MEK**	**MIBK**	**i-BuAl**	**BuAc**	**PPA**	**BTA**	**IVA**	**VLA**
1	14	1,486	3.26	1.08	1.12	1.08	1.10	1.22	1.21	1.26	1.20	1.20	1.20	1.12	1.12	1.11	1.23	1.37	1.33	1.28	1.30
2	70	1,430	16.3	5.42	5.58	5.41	5.50	6.12	6.03	6.28	5.99	5.99	5.98	5.58	5.59	5.55	6.14	6.86	6.64	6.41	6.50
3	140	1,360	32.6	10.8	11.2	10.8	11.0	12.2	12.1	12.6	12.0	12.0	12.0	11.2	11.2	11.1	12.3	13.7	13.3	12.8	13.0
4	280	1,220	65.3	21.7	22.3	21.6	22.0	24.5	24.1	25.1	24.0	24.0	23.9	22.3	22.3	22.2	24.5	27.4	26.6	25.6	26.0
5	700	800	163	54.2	55.8	54.1	55.0	61.2	60.3	62.8	59.9	59.9	59.8	55.8	55.9	55.5	61.4	68.6	66.4	64.1	65.0

a PS: Dilution of pure chemical (primary grade chemical) to make 20 mL solution

b 1st L-WS: Dilution of PS to make 20 mL solution

c Analysis volume: 1 μL

**Table 3S:** Comparison of calibration results determined at the start and end of experiments: Response factor (RF), coefficient of determination (R2), and relative standard errors (RSE).

**Order**	**Group**	**Compound**	**RF**				**R^2^**			**RSE^a^ (%)**
	
			**Exp_day 0**	**Exp_day 9**	**Mean**	**CV^b^**	**Exp_day 0**	**Exp_day 9**	**Mean**	
1	Aldehyde	AA	522	497	509.5	3.47	0.9619	0.9698	0.9659	2.52
2		PA	12,017	11,950	11,984	0.40	0.9991	0.9991	0.9991	3.49
3		BA	43,572	43,467	43,520	0.17	0.9963	0.9938	0.9951	1.05
4		IA	66,125	65,836	65,981	0.31	0.9962	0.9932	0.9947	1.93
5		VA	59,322	59,804	59,563	0.57	0.9973	0.9973	0.9973	1.35
	
6	Aromatic	B	131,760	131,280	131,520	0.26	0.9909	0.9930	0.9920	2.06
7		T	168,602	165,819	167,211	1.18	0.9995	0.9995	0.9995	0.83
8		S	188,198	191,709	189,954	1.31	0.9995	0.9997	0.9996	1.32
9		p-X	188,510	184,038	186,274	1.70	0.9997	0.9987	0.9992	0.49
10		m-X	197,068	193,888	195,478	1.15	0.9992	0.9994	0.9993	0.56
11		o-X	198,376	194,140	196,258	1.53	0.9991	0.9991	0.9991	0.73
	
12	Ketone	MEK	48,980	48,566	48,773	0.60	0.9969	0.9987	0.9978	1.79
13		MIBK	117,383	117,646	117,515	0.16	0.9998	0.9985	0.9992	0.85
	
14	Alcohol	i-BuAl	93,667	92,778	93,223	0.67	0.9969	0.9972	0.9971	1.73
	
15	Ester	BuAc	113,114	117,791	115,453	2.86	0.9982	0.9973	0.9978	0.79
	
16	Carboxyl	PPA	25,574	26,963	26,269	3.74	0.9977	0.9953	0.9965	1.68
17		BTA	71,259	67,832	69,546	3.48	0.9963	0.9967	0.9965	0.13
18		IVA	99,441	94,589	97,015	3.54	0.9965	0.9935	0.9950	2.09
19		VLA	79,615	78,950	79,283	0.59	0.9918	0.9925	0.9922	0.97

		Mean				1.46			0.9954	1.39
		SD				1.29			0.0075	0.82

a Five replicate analyses of 26.1 ng (mean mass) of analytes per 1 μL injection of F-WS (4th calibration point)

b CV (coefficient of variation) = SD/mean * 100

**Table 4S:** Comparison of RF values between actual experiment and the effective carbon number (ECN) approach.

**Order**	**Group**	**Compounds**	**Short name**	**Number of atom and functional group^a^**						**ECN^c^**	**RF values**		**PD^e^**

				**CI^b^**	**HJ^b^**	**OK^b^**	**(>C=O)L^b^**	**(-O-)M^b^**	**(-CH_3_)N^b^**		**Actucal exp**	**ECN approach^d^**	
1	Aldehyde	Acetaldehyde	AA	2	4	1	1	0	1	1.06	510	-15,532	
2		Propionaldehyde	PA	3	6	1	1	0	1	1.99	11,984	12,921	7.82
3		Butyraldehyde	BA	4	8	1	1	0	1	2.92	43,520	41,375	4.93
4		Isovaleraldehyde	IA	5	10	1	1	0	2	4.00	65,981	74,418	12.8
5		n-Valeraldehyde	VA	5	10	1	1	0	1	3.85	59,563	69,828	17.2
	
6	Aromatic	Benzene	B	6	6	0	0	0	0	5.79	131,520	129,183	1.78
		Toluene	T	7	8	0	0	0	1	6.87	167,211	162,226	2.98
8		Styrene	S	8	8	0	0	0	0	7.72	189,954	188,232	0.91
9		p-Xylene	p-X	8	10	0	0	0	2	7.95	186,274	195,269	4.83
10		m-Xylene	m-X	8	10	0	0	0	2	7.95	195,478	195,269	0.11
11		o-Xylene	o-X	8	10	0	0	0	2	7.95	196,258	195,269	0.50
	
12	Ketone	Methyl ethyl ketone	MEK	4	8	1	1	0	2	3.07	48,773	45,964	5.76
13		Methyl isobutyl ketone	MIBK	6	12	1	1	0	3	5.08	117,515	107,460	8.56
	
14	Alcohol	Isobutyl alcohol	i-BuAl	4	10	1	0	1	2	4.50	93,223	89,715	3.76
	
15	Ester	n-Butyl acetate	BuAc	6	12	2	1	1	2	5.48	115,453	119,698	3.68
	
16	Fatty acid	Propionic acid	PPA	3	6	2	1	1	1	2.54	26,269	29,748	13.2
17		n-Butyric acid	BTA	4	6	2	1	1	1	3.54	69,546	60,344	13.2
18		i-Valeric acid	IVA	5	10	2	1	1	2	4.55	97,015	91,245	5.95
19		n-Valeric acid	VLA	5	10	2	1	1	1	4.40	79,283	86,656	9.30

											Mean:6.52 ± 4.99		

a Carbon number equivalent for each atom and functional group (CNE): (1) C = 1, (2) H = −0.035, (3) O = 0, (4) >C=0 = −0.95, (5) -O- = 0.55, and (6) − CH3 = 0.15

b I, J, K, L, M and N = number of C, H, O, >=O, -O-, CH3 (atoms or functional groups) in each VOC, respectively

c ECN = I + J*(CNE of -H) + L*(CNE of >C=O) + M*(CNE of -O-) + N*(CNE of -CH3)

d The predictive equation (by ECN approach) for estimation of VOC concentration was determined using 18 liquid working standards except for AA: (1) RF = 30,595, (2) intercept = -47,963, and (3) R2 = 0.9901

e Percent different (PD, %) = ABS{[RF (actual exp)-RF (ECN approach)] / RF (actual exp) * 100}

**Table 5S:** Operational conditions of TD-GC-TOF MS system for the analysis of fresh and decaying strawberry.

[A] Sampling information of strawberry volatiles			

**a. Information of strawberry for sampling**			

**Order**	**Sample code**	**Storage time (day)**	**Storage temp. (°C )**
	
1	SRD-0	0	25
2	SRD-1	1	25
3	SRD-3	3	25
4	SRD-6	6	25
5	SRD-9	9	25

Initial weight = 50.09 g			

b. Sampling approach	

Sampler:	3 bed sorbent tube
Purge gas:	Nitrogen (>99.999%)
Purge gas flow:	50 mL·min^−1^
Pump model:	MP-Σ30 (Sibata, Japan)
Heater model:	TC200P (Korea)

[B] Instrumental setups for VOC analysis			

**a. GC (Shimadzu GC-2010, Japan) and MS (Shimadzu GCMS-QP2010, Japan) Column: CP Wax (diameter: 0.25 mm, length: 60 m, and film thickness: 0.25 μm )**			

**Oven setting**		**Detector setting**	
	
Initial temp:	35 °C (10 min)	Ionization mode:	EI (70 eV)
Ramp rate:	6 °C·min ^−1^	Ion source temp.:	200 °C
Max oven temp:	215 °C (10 min)	Interface temp.:	200 °C
Total time:	50 min	TIC scan range:	35∼260 m/z
Carrier gas:	He (99.999%)		
Carrier gas flow:	1 mL·min ^−1^		

**b. Thermal desorber (Unity, Markes, UK)**			

Cold trap sorbent:	Tenax TA + Carbopack B (volume ratio=1:1) (diameter: 2 mm and sorbent bed length = 5 mm)		
Split ratio:	1:5	Adsorption temp.:	-10 °C
Split flow:	5 mL·min^−1^	Desorption temp.:	320 °C
Trap hold time:	20 min	Flow path temp:	150 °C

**c. Sorbent (Sampling) Tube**			

Sorbent material:	Tenax TA + Carbopack B + Carboxen 1000 (mass (mg)=100 : 100: 100 )		
Desorption flow:	50 mL·min^−1^		
Desorption time:	5 min	Desorption temp.:	300 °C

**Table 6S:** A list of individual VOC determined by the TD-GC-MS system from all strawberry samples throughout the study period.

**Order**	**Compounds**	**MW**	**Formula**	**Concentration**										**Odor threshold**			**Averaged Similarity * (%)**
	
				**ppb**					**(μg m^−3^)**								
		
		**(g mole^−1^)**		**SRD-0**	**SRD-1**	**SRD-3**	**SRD-6**	**SRD-9**	**SRD-0**	**SRD-1**	**SRD-3**	**SRD-6**	**SRD-9**	**(ppbv)**	***(*μg m^3^)**	**Reference**	
**A. Ester (61)**																	
1	Methyl acetate	74	C3H6O2	3,949	3,875	71,077	1.57	0.75	11,945	11,720	214,987	4.76	2.28	1,700-6,170	5,142-	b, c	98.4
2	Ethyl acetate	88	C4 H8 O2	4,793	4,277	134,969	130.0	11.80	17,240	15,384	485,476	467	42.5	610-329	1,183-9,460	a, b, c, e	98.8
3	Methyl propionate	88	C4H8O2	40.8	51.0	23.6	0.17	0.009	147	183	85.0	0.63	0.031	98	352	b	97.8
4	S-Methyl thioacetate	90	C3 H6 O S	94.2	130	0.031	0.031	0.031	346	475.8	0.113	0.113	0.113	1.6	5.9	a	97.0
5	Methyl trans-crotonate	100	C5 H8 O2	2.77	3.16	8.51	0.005	0.005	11.3	12.9	34.8	0.021	0.021				92.0
6	Isopropyl acetate	102	C5H10O2	52.8	16.7	27.5	0.005	0.005	220	69.5	114.5	0.021	0.021	290-2,400	1,209-	a, b, c	98.3
7	Methyl isobutyrate	102	C5H10O2	0.005	0.005	0.005	0.43	0.005	0.021	0.021	0.021	1.77	0.021	1.9	10,006 7.9	b	91.0
8	Ethyl propionate	102	C5 H10 O2	52.7	0.005	53.5	0.005	0.005	219.8	0.022	223	0.022	0.022	7	29	b	98.5
9	Propyl acetate	102	C5 H10 O2	0.005	0.005	185	0.72	0.005	0.022	0.022	769	3.02	0.022	568-960	2,367-4,002	a, b, c	97.5
10	Methyl butyrate	102	C5 H10 O2	1194	1,384	88.8	0.47	0.005	4,977	5,772	370	1.95	0.022	2.8-7.1	12-30	a, b	98.0
11	Ethyl crotonate	114	C6 H10 O2	7.13	4.49	26.2	0.003	0.003	33.22	20.91	122	0.016	0.016				95.7
12	Methyl tiglate	114	C6H10O2	0.003	0.003	1.26	0.003	0.003	0.016	0.016	5.88	0.016	0.016				95.0
13	Ethyl isobutyrate	116	C6 H12 O2	3.80	1.29	8.57	0.86	0.003	18.0	6.11	40.6	4.07	0.016	0.22	0.10	b	95.0
14	Methyl 2-methylbutanoate	116	C6H12O2	21.8	21.7	13.2	1.08	0.003	104	103	62.7	5.14	0.016	0.1	0.5	a	97.3
15	Isobutyl acetate	116	C6H12O2	9.11	7.00	88.9	1.86	0.003	43.2	33.2	421	8.80	0.016	8-479	38-2,271	b, c	98.0
16	Methyl isovalerate	116	C6 H12 O2	54.8	56.9	7.78	0.003	0.003	260	270	36.9	0.016	0.016	1.1-2.2	5.2-10	a, b	97.7
17	Ethyl butyrate	116	C6 H12 O2	1,537	1,142	171	1.50	0.003	7,290	5,415	812	7.10	0.017	0.04-0.2	0.19-1.2	a, b	97.5
18	*Butyl acetate*	116	C6 H12 O2	34.2	36.5	26.4	0.05	0.003	162	173	125	0.24	0.017	10.7-195	50.9-925	a, b, c	93.0
19	Methyl valerate	116	C6 H12 O2	3.91	1.74	0.003	0.003	0.003	18.5	8.26	0.017	0.017	0.017	2.2	10	b	95.5
20	S-Methyl thiobutyrate	118	C5H10OS	0.006	4.47	0.006	0.006	0.006	0.027	21.57	0.027	0.027	0.027				92.0
21	Methyl 2-vinylbutanoate	128	C7 H12 O2	0.77	0.003	0.003	0.003	0.003	4.03	0.013	0.013	0.013	0.013				96.0
22	4-Penten-1-yl acetate	128	C7 H12 O2	2.65	0.80	3.74	0.003	0.003	13.8	4.20	19.6	0.014	0.014				91.3
23	Ethyl tiglate	128	C7H12O2	0.002	1.41	16.6	0.002	0.002	0.013	7.40	86.7	0.013	0.013				94.5
24	cis-2-Penten-1-yl acetate	128	C7 H12 O2	4.96	0.003	0.003	0.003	0.003	25.95	0.013	0.013	0.013	0.013				91.0
25	Prenyl acetate	128	C7H12O2	0.48	2.83	0.002	0.002	0.002	2.52	14.81	0.013	0.013	0.013				96.0
26	Methyl (2E)-2-hexenoate	128	C7H12O2	1.66	0.89	0.66	0.003	0.003	8.66	4.68	3.44	0.013	0.013				
27	Isopropyl butyrate	130	C7H14O2	30.7	25.9	0.002	0.002	0.002	163.0	137.8	0.013	0.013	0.013	6.2	33	b	93.5
28	Ethyl 2-methylbutyrate	130	C7H14O2	63.0	23.8	66.3	3.13	0.002	335	126	352	16.6	0.013	0.07	0.36	a	97.0
29	Ethyl isovalerate	130	C7 H14 O2	142	67.2	36.1	0.70	0.002	753	357	192	3.73	0.013	0.013-0.09	0.069-0.47	a, b	96.3
30	Isoamyl acetate	130	C7 H14 O2	81.3	69.1	165	1.21	0.002	432	367.15	878	6.43	0.013	3.8	20	e	98.0
31	Ethyl valerate	130	C7 H14 O2	7.31	1.73	0.003	0.003	0.003	38.83	9.22	0.013	0.013	0.013	0.11-0.33	0.58-1.78	a, b	96.5
32	Methyl 4-methylvalerate	130	C7H14O2	2.58	0.96	0.002	0.002	0.002	13.72	5.09	0.013	0.013	0.013				94.5
33	Methyl hexanoate	130	C7 H14 O2	469	185	11.36	0.003	0.003	2,492	982	60.34	0.013	0.013	15.6	82.8	a	97.0
34	3-Methyl-2-butyl acetate	130	C7 H14 O2	0.002	0.002	0.48	0.002	0.002	0.013	0.013	2.55	0.013	0.013				93.0
35	Hex-5-enoic acid, ethyl ester	142	C8H14O2	1.31	0.002	0.002	0.002	0.002	7.62	0.011	0.011	0.011	0.011				92.0
36	cis-3-Hexenyl acetate	142	C8 H14 O2	2.43	0.002	0.002	0.002	0.002	14.1	0.011	0.011	0.011	0.011	3.3	19	a	95.0
37	(4E)-4-Hexenyl acetate	142	C8H14O2	5.41	2.68	0.002	0.002	0.002	31.4	15.5	0.011	0.011	0.011				91.0
38	trans-2-Hexenyl acetate	142	C8 H14 O2	38.6	1.78	0.002	0.002	0.002	224	10.4	0.011	0.011	0.011	42.8	249	a	92.0
39	Ethyl 2-hexenoate	142	C8H14O2	3.29	1.24	0.002	0.002	0.002	19.1	7.21	0.011	0.011	0.011				95.5
40	Methyl amyl acetate	144	C8 H16 O2	0.002	0.002	0.37	0.002	0.002	0.011	0.011	2.16	0.011	0.011				93.0
41	Isobutyl butyrate	144	C8H16O2	0.002	1.13	0.91	0.002	0.002	0.011	6.65	5.35	0.011	0.011	1.6	9.4	b	93.5
42	Propyl isovalerate	144	C8H16O2	0.39	0.48	0.75	0.002	0.002	2.29	2.85	4.40	0.011	0.011	0.056	0.330	b	91.0
43	Butyl butylate	144	C8 H16 O2	0.78	1.37	0.002	0.002	0.002	4.60	8.08	0.011	0.011	0.011	4.8-22.1	28-130	a, b	96.5
44	Ethyl hexanoate	144	C8H16O2	725	137	20.9	0.03	0.002	4,270	808	123	0.16	0.011	0.2	1.2	a	97.0
45	Hexyl acetate	144	C8H16O2	110	25.5	4.70	0.002	0.002	647	150	27.7	0.011	0.011	2	12	a	97.7
46	Valeric acid, thio-, S-ethyl ester	146	C7 H14 O S	0.89	0.003	0.003	0.003	0.003	5.29	0.015	0.015	0.015	0.015				95.0
47	Ethyl 3-hydroxy-3-methylbutanoate	146	C7 H14 O3	0.002	0.002	0.002	0.04	0.002	0.012	0.012	0.012	0.21	0.012				97.0
48	Ethyl benzoate	150	C9H10O2	0.39	0.002	0.002	0.02	0.002	2.37	0.009	0.009	0.15	0.009				90.0
49	Isobutyl isovalerate	158	C9H18O2	0.001	0.001	0.33	0.001	0.001	0.009	0.009	2.15	0.009	0.009	5.2	34	b	94.0
50	Neopentyl butyrate	158	C9H18O2	0.001	0.59	0.001	0.001	0.001	0.009	3.78	0.009	0.009	0.009				93.0
51	Isoamyl butyrate	158	C9 H18 O2	1.58	1.53	0.001	0.001	0.001	10.2	9.87	0.009	0.009	0.009				94.0
52	Heptyl acetate	158	C9H18O2	0.29	0.001	0.001	0.001	0.001	1.85	0.010	0.010	0.010	0.010				91.0
53	Methyl octanoate	158	C9 H18 O2	5.84	0.88	0.001	0.001	0.001	37.7	5.710	0.010	0.010	0.010	36.7	237	a	95.5
54	Octyl acetate	172	C10 H20 O2	0.55	0.001	0.001	0.001	0.001	3.86	0.008	0.008	0.008	0.008				95.0
55	Hexyl butyrate	172	C10H20O2	0.52	0.001	0.001	0.001	0.001	3.65	0.008	0.008	0.008	0.008				92.0
56	Ethyl octanoate	172	C10H20O2	4.55	0.001	0.001	0.001	0.001	32.0	0.008	0.008	0.008	0.008				94.0
57	Octyl acetate	172	C10H20O2	1.25	0.001	0.001	0.001	0.001	8.80	0.008	0.008	0.008	0.008				91.0
58	Cyclopentanecarboxylic acid, decyl ester	254	C16H30O2	0.0005	0.0005	0.0005	0.0005	0.0555	0.0049	0.0049	0.0049	0.005	0.58				92.0
59	2-(Dodecyloxy)ethyl acetate	272	C16H32O3	0.0004	0.0004	0.11	0.0004	0.0004	0.0047	0.0047	1.21	0.005	0.0047				92.0
60	Linalylanthranilate N-(tert-butoxycarbonyl)-glycyl-	273	C17 H23 N O2	0.44	0.0004	0.0004	0.0004	0.0004	4.95	0.0044	0.0044	0.004	0.0044				93.0
61	glycin-imidthiosaure-s-ethylester	275	C11H21N3O3S	0.001	0.001	0.001	0.05	0.03	0.008	0.008	0.008	0.58	0.39				92.5

	Σconcentration (ester)								52,648	42,713	705,447	533	45.7				
**B. Alcohol (21)**																	
62	Ethyl alcohol	46	C2H6O	0.037	0.037	309	48,685	1,417	0.070	0.070	580	91,537	2,665	520-28,800	978-54,150	b, c	95 .6
63	Isopropyl Alcohol	60	C3H8O	8.76	4.58	45.3	92.8	278	21.5	11.2	111	228	680.73	10,200-	25,015-	b, c	95.6
64	n-Propyl alcohol	60	C3 H8 O	0.014	0.014	30.40	46.6	36.1	0.035	0.035	74.54	114.4	88.51	94-2,400	231-5,886	b, c	94.3
65	sec-Butyl alcohol	74	C4 H10 O	0.007	0.007	0.007	0.56	0.55	0.022	0.022	0.022	1.70	1.65	220-1,700	665-5,142	b, c	93.5
66	*Isobutyl alcohol*	74	C4 H10 O	0.007	0.007	31.84	95,136	319.8	0.021	0.021	96.30	287,758	967	11-832	33-2,517	b, c	97.7
67	n-Butyl alcohol	74	C4H10O	0.008	2.40	8.78	0.86	0.37	0.023	7.26	26.6	2.61	1.12	490-26,000	1,482-	b, c	96.3
68	1-Penten-3-ol	86	C5 H10 O	0.005	0.005	0.61	0.005	0.005	0.017	0.017	2.13	0.017	0.017				94.0
69	Isopropenylethyl alcohol	86	C5H10O	0.005	0.005	0.005	2.41	4.47	0.017	0.017	0.017	8.48	15.7	2,500	8,788	e	96.0
70	3-Methyl-1-butanol	88	C5 H12 O	1.09	1.28	50.7	26.9	43.4	3.91	4.61	182.26	96.9	156	2-45	6-161	b, c	93.2
71	2-Methyl-1-butanol	88	C5 H12 O	0.005	0.005	0.005	9.48	19.6	0.017	0.017	0.017	34.12	70.50	41	147	e	98.0
72	n-Pentanol	88	C5 H12 O	0.005	0.005	0.005	0.99	0.86	0.017	0.017	0.017	3.57	3.10	100-468	360-1,683	b, c	96.0
73	1-Chloro-2-propanol	94	C3 H7 CL O	20.03	80.3	0.009	0.009	0.009	77.0	309	0.034	0.034	0.034	1,000	3,842	f	93.0
74	3-Methylpentanol	102	C6 H14 O	0.003	0.003	0.003	0.05	0.34	0.013	0.013	0.013	0.22	1.41				93.0
75	n-Hexanol	102	C6 H14 O	0.52	0.003	1.16	0.15	1.08	2.17	0.014	4.83	0.63	4.51	1.1-43.7	4.6-182	b, c, e	92.8
76	2-Heptanol	116	C7 H16 O	0.002	0.002	0.002	0.42	0.17	0.011	0.011	0.011	2.01	0.81				93.5
77	Phenethyl alcohol	122	C8 H10 O	0.002	0.002	0.002	0.15	0.13	0.010	0.010	0.010	0.73	0.65				92.0
78	(E)-2-Octen-1-ol	128	C8H16O	0.25	0.002	0.002	0.002	0.002	1.31	0.010	0.010	0.010	0.010				
79	1-Octen-3-ol	128	C8 H16 O	0.002	0.002	0.002	0.02	0.12	0.010	0.010	0.010	0.09	0.62	2.26	11.8	a	91.0
80	2-Ethylhexanol	130	C8 H18 O	0.42	0.002	0.002	0.05	0.12	2.21	0.010	0.010	0.28	0.63				91.0
81	Linalool	154	C10 H18 O	5.14	5.08	0.001	0.001	0.001	32.4	32.0	0.007	0.007	0.007	0.19	1.18	a	93.0
82	.alpha.-Methyl-.alpha.-[4-methyl-3-pentenyl]oxiranemethanol	170	C10H18O2	0.001	0.001	0.001	0.03	0.001	0.007	0.007	0.007	0.17	0.007		91.0

			Sum						140	364	1,078	379,789	4,658				
**C. Aldehyde (11)**																	
83	*Acetaldehyde*	44	C2 H4 O	1,618	1,646	10,584	730	540	2,909	2,959	19,035	1,313	971	1.5-186	2.7-335	b, c	99 .2
84	Allyl aldehyde (Acrolein)	56	C3 H4 O	0.082	0.082	0.082	5.08	6.90	0.189	0.189	0.189	11.62	15.8	3.6-174	8.2-398	b, c	98.0
85	Methylacryl aldehyde	70	C4 H6 O	0.016	0.016	0.016	1.80	3.33	0.045	0.045	0.045	5.14	9.52	8.5	24	b	
86	Isobutyraldehyde	72	C4H8O	0.015	0.015	0.015	0.24	3.80	0.043	0.043	0.043	0.71	11.17	0.35-40.7	1.03-120	b, c	
87	*Butyraldehyde*	72	C4H8O	0.015	0.015	0.015	0.35	0.015	0.045	0.045	0.045	1.04	0.045	0.67-8.91	1.97-26.2	b, c	96.0
88	Methylethylacetaldehyde	86	C5H10O	0.008	0.008	0.008	0.15	0.51	0.027	0.027	0.027	0.52	1.79				94.5
89	*Isovaleraldehyde*	86	C5 H10 O	0.009	0.009	0.009	1.03	2.45	0.030	0.030	0.030	3.60	8.62	0.1-2.24	0.4-7.87	b, c	96.0
90	n-Caproaldehyde	100	C6H12O	1.38	0.005	0.77	0.005	0.005	5.66	0.020	3.14	0.020	0.020	0.28-13.8	1.14-56.4	a, b, c	91.0
91	Benzaldehyde	106	C7 H6 O	0.003	0.003	3.71	0.50	0.22	0.015	0.015	16.1	2.16	0.97	41.7	181	c	94.3
92	n-Nonylaldehyde	142	C9 H18 O	0.55	0.002	0.002	0.002	0.002	3.17	0.011	0.011	0.011	0.011	0.34-2.24	1.97-13.0	b, c	91.0
93	2,4-dihydroxy-6-(2′-oxoheptyl)benzaldehyde	250	C14 H18 O4	0.001	0.001	0.001	0.001	0.36	0.006	0.006	0.006	0.006	3.64				96.0

			Sum						2,918	2,959	19,054	1,338	1,022				
**D. Ketone (9)**																	
94	Acetone	58	C3 H6 O	2,514	3,131	224	7.35	28.0	5,960	7,422	532	17.43	66.5	4,580-42,000	10,858-	b, c, e	97.8
95	Methyl vinyl ketone	70	C4 H6 O	0.016	0.016	1.25	0.016	0.016	0.045	0.045	3.57	0.045	0.045	183	524	d	91.0
96	*Methyl ethyl ketone*	72	C4 H8 O	0.014	0.014	0.014	1.15	1.35	0.041	0.041	0.041	3.39	3.96	270-7,760	795-22,837	b, c, e	93.0
97	Methyl n-propyl ketone	86	C5H10O	54.0	47.1	0.008	0.008	0.008	190	166	0.027	0.027	0.027	28-1,550	98-5,449	b, c	93.5
98	Dimethyl diketone	86	C4H6O2	13.7	0.030	13.9	3.21	0.030	48.10	0.104	48.89	11.3	0.104	4.37	15.4	c	96.3
99	*Methyl Isobutyl Ketone*	100	C6H12O	4.62	7.50	1.11	0.004	0.004	18.87	30.7	4.53	0.017	0.017	121-537	495-2,195	b, c, e	95.0
100	Methyl amyl ketone	114	C7 H14 O	5.64	6.52	0.003	0.51	0.61	26.28	30.4	0.015	2.36	2.85	35.6-141	166-657	a, b, c	97.8
101	Spiro[3.6]deca-5,7-diene-1-one	148	C10 H12 O	0.001	0.001	0.15	0.001	0.001	0.009	0.009	0.91	0.009	0.009				94.0
102	Phenyl methyl ketone	120	C8 H8 O	1.56	0.002	0.002	0.10	0.13	7.67	0.012	0.012	0.50	0.62	363	1,780	c	91.3
	
			Sum						6,251	7,649	589	35	74				
**E. Fatty acid (5)**																	
103	Acetic acid	60	C2H4O2	2,519	1,918	1,761	210	86	6,177	4,703	4,319	514	212	6-145	15-356	b, c, e	93 .0
104	2-Propynoic acid	70	C3 H2 O2	69.6	0.023	0.023	0.023	0.023	199	0.067	0.067	0.067	0.067	94	269	i	93.0
105	Methacrylic acid	86	C4H6O2	3.92	0.009	0.009	0.009	0.009	13.8	0.033	0.033	0.033	0.033				93.0
106	2-Methylbutanoic acid	102	C5 H10 O2	0.005	0.005	0.005	0.31	0.005	0.022	0.022	0.022	1.30	0.022	8-186	33-775	a, c, e	93.0
107	Undec-10-ynoic acid	182	C11H18O2	0.001	0.001	0.001	0.001	0.04	0.008	0.008	0.008	0.008	0.28				92.0
	
			Sum						6,390	4,703	4,319	516	212				
**F.*etc.* (40)**																	
108	Isoprene	68	C5 H8	3.98	6.11	4.08	0.007	0.77	11.06	17.0	11.34	0.020	2.13	48-455	133-1,265	b, e	95.3
109	Ethylidenecyclopropane	68	C5 H8	0.007	0.007	0.007	0.06	0.007	0.020	0.020	0.020	0.17	0.020				93.0
110	cis-Piperylene	68	C5 H8	1.54	1.49	0.007	0.17	0.007	4.29	4.14	0.020	0.46	0.020				89.3
111	Methylenecyclobutane	68	C5H8	0.007	0.79	0.54	0.007	0.007	0.020	2.20	1.49	0.020	0.020				84.5
112	Divinylene oxide	68	C4H4O	0.008	0.008	0.008	0.008	0.60	0.023	0.023	0.023	0.023	1.66				97.0
113	Propylethylene	70	C5 H10	0.007	0.007	0.74	0.007	0.007	0.020	0.020	2.12	0.020	0.020				90.0
114	n-Pentane	72	C5 H12	12.1	0.007	0.007	0.29	2.20	35.7	0.019	0.019	0.86	6.48	1,400-31,600	4,120-92,997	b, c	96.3
115	Furanidine	72	C4H8O	0.008	0.008	0.008	0.008	0.56	0.024	0.024	0.024	0.024	1.65		97.0
116	Ethyl ether	74	C4 H10 O	0.007	0.007	0.70	0.41	1.45	0.022	0.022	2.12	1.23	4.38	330	998	h	93.7
117	1,3-Hexadiene	82	C6 H10	25.0	1.77	0.005	0.005	0.005	83.9	5.94	0.015	0.015	0.015	2,000	6,703	g	97.0
118	Furan, 2-methyl-	82	C5 H6 O	0.005	0.005	0.005	0.005	0.60	0.016	0.016	0.016	0.016	2.00	24,678	82,713	d	93.0
119	Methylcyclopentane	84	C6H12	0.005	0.005	0.005	0.005	0.57	0.016	0.016	0.016	0.016	1.97				96.0
120	n-Hexane	86	C6H14	1.21	1.02	1.02	0.004	8.03	4.27	3.60	3.57	0.015	28.22	1,500-21,900	5,273-76,983	b, c	94.3
121	2,2-Dimethylbutane	86	C6 H14	3.49	0.004	0.004	0.004	0.004	12.27	0.014	0.014	0.014	0.014		90.0
122	*Toluene*	92	C7H8	0.003	0.003	0.003	0.003	2.48	0.012	0.012	0.012	0.012	9.32	160-1,550	602-5,829	b, c, e	97.0
123	Phenol	94	C6H6O	0.004	0.004	0.004	0.22	0.004	0.014	0.014	0.014	0.84	0.014	5.60-110	21.5-423	b, c	91.0
124	1-Heptene	98	C7H14	0.42	0.63	0.47	0.17	0.56	1.69	2.51	1.87	0.68	2.24				94.4
125	3-Methylhexane	100	C7H16	0.003	0.003	0.003	0.003	0.08	0.012	0.012	0.012	0.012	0.33	840	3,433	b	92.0
126	*Styrene*	104	C8 H8	10.05	11.99	19.41	3.03	1.09	42.7	51.0	82.5	12.9	4.64	34.4-35	146-149	b, e	93.2
127	Ethylbenzene	106	C8H10	1.65	0.002	0.002	0.002	0.10	7.16	0.010	0.010	0.010	0.44	2.88-170	12.5-737	b, c	94.5
128	*p-Xylene*	106	C8 H10	0.32	0.002	0.002	0.002	0.02	1.39	0.011	0.011	0.011	0.10	58-490	251-2,123	b, c	93.0
129	3-Cyclohexenyl cyanide	107	C7 H9 N	0.003	0.003	0.003	0.003	0.13	0.013	0.013	0.013	0.013	0.55				92.0
130	Anisole	108	C7H8O	0.002	0.002	0.002	0.06	0.002	0.011	0.011	0.011	0.27	0.011				90.0
131	n-Octane	114	C8 H18	0.002	0.002	0.002	0.002	0.12	0.011	0.011	0.011	0.011	0.57	1,700-5,750	7,921-26,793	b, c	91.0
132	p-Allyltoluene	132	C10H12	0.52	0.001	0.001	0.001	0.001	2.78	0.008	0.008	0.008	0.008				94.0
133	p-Isopropylphenol	136	C9H12O	0.001	0.001	0.001	0.001	0.04	0.008	0.008	0.008	0.008	0.23				93.0
134	2-Methyl-6-methylene-2,7-octadiene	136	C10 H16	0.001	0.42	0.001	0.001	0.001	0.008	2.34	0.008	0.008	0.008				92.0
135	l-Limonene	136	C10 H16	0.25	0.52	0.001	0.001	0.001	1.36	2.88	0.008	0.008	0.008				90.0
136	(E)-.beta.-Ocimene	136	C10 H16	0.24	0.001	0.001	0.001	0.001	1.33	0.008	0.008	0.008	0.008				90.0
137	Tetramethylbutanedinitrile	136	C8H12N2	0.59	0.002	0.002	0.002	0.002	3.30	0.010	0.010	0.010	0.010				94.0
138	2,2,3,3-Tetramethylhexane	142	C10 H22	0.001	1.65	0.001	0.001	0.001	0.008	9.57	0.008	0.008	0.008				94.0
139	2,5-Dimethyl-4-methoxy-3(2H)-furanone	142	C7H10O3	1.42	0.002	0.002	2.66	0.88	8.23	0.011	0.011	15.5	5.08	3.26	18.9	a	91.7
140	m-Dichlorobenzene	146	C6H4Cl2	0.003	0.003	0.003	0.06	0.08	0.015	0.015	0.015	0.34	0.46				96.5
141	3,5-Dihydroxybenzamide	153	C7H7NO3	0.002	0.002	0.002	0.03	0.002	0.012	0.012	0.012	0.18	0.012				91.0
142	n-Butanoic anhydride	158	C8H14O3	0.002	0.53	0.002	0.002	0.002	0.013	3.44	0.013	0.013	0.013				93.0
143	Genitron	164	C8H12N4	0.001	0.001	0.001	0.05	0.001	0.010	0.010	0.010	0.33	0.010				90.0
144	2-Propyloctahydro-2H-thiochromene	198	C12H22S	0.001	0.001	0.001	0.01	0.001	0.007	0.007	0.007	0.04	0.007				90.0
145	l-Caryophyllene	204	C15 H24	0.001	0.001	0.001	0.15	0.11	0.005	0.005	0.005	1.28	0.88				92.0
146	.alpha.-Muurolene	204	C15 H24	0.001	0.001	0.001	0.01	0.01	0.005	0.005	0.005	0.11	0.11				91.0
147	2,3-Epoxy-.beta.-ionone	208	C13 H20 O2	0.001	1.28	0.001	0.001	0.001	0.006	10.9	0.006	0.006	0.006				98.0

			Sum						221	115	105	35.1	73.4			Mean	94.1
			Total						68,569	58,503	730,593	382,245	6,086				

Below detection limit

References: a. Van Gemert [50], b. Nagata [26], c. Devos *et al.* [51], d. Ruth [52], e. Woodfield and Hall [53], f. Chemwatch [54], g. Evans *et al.* [55], h. U.S. Department of Labor [56], and i. Amoore and Hautala [57].

* Mean similarity of mass spectra between actual experiment and library (NIST)

**Table 7S:** Concentrations of reduced sulfur compounds (RSC) and ammonia measured separately by GC-PFPD and absorption photometry.

**Order**	**Compounds**	**SRD-0**	**SRD-1**	**SRD-3**	**SRD-6**	**SRD-9**	**Thresholds^b^ (μg m^−3^)**	**Reference**
**[A] Concentration (μg m^−3^)**								

1	Hydrogen sulfide	0.02^a^	0.02	0.02	0.02	0.02	24.8	Schiffman *et al.* [58]
2	Sulfur dioxide	0.03	0.03	0.03	0.03	0.03	1,635	Nagata [26]
3	Methanethiol	61.4	267	0.79	0.02	0.49	4.13	Iowa State University [59]
4	Dimethyl sulfide	0.02	3.83	0.91	0.02	0.02	7.60	Nagata [26]
5	Dimethyl disulfide	18.9	196	35.8	0.57	0.79	47.3	Schiffman *et al.* [58]
6	Ammonia	81.3	81.3	81.3	169	445	1,042	Nagata [26]
**[B] Odor activity value (OAV: concentration/ threshold)**								
	
1	Hydrogen sulfide							
2	Sulfur dioxide							
3	Methanethiol	14.9	64.6	0.19		0.12		
4	Dimethyl sulfide		0.50	0.12				
5	Dimethyl disulfide	0.40	4.14	0.76	0.01	0.02		
6	Ammonia				0.16	0.43		

a Values below detection limit are underlined (calculated as method detection limit)

**Table 8S:** The basic statistics on threshold values^a^ (n = 79) of VOC.

[A] Number of contrasting sources for the VOC with odor thresholds						

No. of sources for odor thresholds	0 (no data)	1	2	3	4	Total
No. of VOC	73	35	31	12	1	152

[B] The basic statistics of threshold values of VOCs (n = 79)							

**Statistical parameter**	**Mean**	**SD**	**CV b**	**Minimum**	**Maximum**	**Median**	**Sum**

Minimum	1,929	8,272	429	0.058	69,896	28	152,379
Maximum	7,576	19,199	253	0.088	84,140	227	598,518
Geo mean	3,077	9,552	310	0.088	69,896	123	243,085

a We determined the 79 threshold values out of the 147 VOCs detected from strawberry samples from previous studies(references from Table 5S)

b CV (coefficient of variation) = SD/mean × 100

**Table 9S:** Log normal relationship between different types of odor threshold (ppbv) and molecular weight to assess the odor strength patterns of strawberry volatiles.

**Groups**	**A. Ester**		**B. Alcohol**	**C. Aldehyde**		**D. Ketone**		**Sum of all groups b**	
				
	**All data**	**Optimal fit a**	**All data**	**All data**	**Optimal fit**	**All data**	**Optimal fit**	**All data**	**Optimal fit**
**Number of data**	(n = 31)	(n = 28)	(n = 14)	(n = 9)	(n = 6)	(n = 8)	(n = 6)	(n = 62)	(n = 54)
**[A] Slope** c									

Min	−0.0237	−0.0336	−0.0357	−0.0043	−0.0086	−0.0152	−0.0171	−0.0222	−0.0256
Max	−0.0296	−0.0406	−0.0507	−0.0161	−0.0187	−0.0253	−0.0366	−0.0351	−0.0415
Geo mean	−0.0263	−0.0367	−0.0432	−0.0098	−0.0142	−0.0197	−0.0268	−0.0286	−0.0329

**[B] R^2^**									

Min	0.1511	0.2842	0.5048	0.0231	0.4825	0.1360	0.3959	0.2171	0.2897
Max	0.1906	0.3363	0.8397	0.4396	0.8833	0.2113	0.9237	0.4260	0.5743
Geo mean	0.1743	0.3188	0.7164	0.1934	0.8730	0.1885	0.6547	0.3384	0.4473

**[C] p-value**									

Min	3.07E-02	3.49E-03	4.40E-03	6.96E-01	1.26E-01	3.69E-01	1.81E-01	1.35E-04	2.69E-05
Max	1.41E-02	1.22E-03	4.12E-06	5.16E-02	5.32E-03	2.52E-01	2.24E-03	9.00E-09	3.26E-11
Geo mean	1.94E-02	1.75E-03	1.35E-04	2.36E-01	6.33E-03	2.82E-01	5.12E-02	7.10E-07	3.25E-08

a After excluding outlying data points

b Sum of the four major VOC groups (Ester, Alcohol, Aldehyde, and Ketone)

c Results of linear regression analysis between odor threshold and molecular weights: for this comparison, results are compared between minimum, maximum, and geometric mean of threshold values available from previous studies
